# Measuring the Frequency-Specific Functional Connectivity Using Wavelet Coherence Analysis in Stroke Rats Based on Intrinsic Signals

**DOI:** 10.1038/s41598-020-66246-9

**Published:** 2020-06-10

**Authors:** Leila Mohammadzadeh, Hamid Latifi, Sepideh Khaksar, Mohammad-Sadegh Feiz, Fereshteh Motamedi, Amir Asadollahi, Marzieh Ezzatpour

**Affiliations:** 1 Laser and Plasma Research Institute, Shahid Beheshti University, Tehran, 1983969411 Iran; 2 Department of Physics, Shahid Beheshti University, Tehran, 1983963113 Iran; 3 Department of Plant Sciences, Faculty of Biological Sciences, Alzahra University, Tehran, 1993893973 Iran; 4 Neuroscience Research Center, Shahid Beheshti University of Medical Sciences, Tehran, 1983963113 Iran

**Keywords:** Biophysics, Neuroscience, Diseases, Optics and photonics

## Abstract

Optical intrinsic signal imaging (OISi) method is an optical technique to evaluate the functional connectivity (FC) of the cortex in animals. Already, using OISi, the FC of the cortex has been measured in time or frequency domain separately, and at frequencies below 0.08 Hz, which is not in the frequency range of hemodynamic oscillations which are able to track fast cortical events, including neurogenic, myogenic, cardiac and respiratory activities. In the current work, we calculated the wavelet coherence (WC) transform of the OISi time series to evaluate the cerebral response changes in the stroke rats. Utilizing WC, we measured FC at frequencies up to 4.5 Hz, and could monitor the time and frequency dependency of the FC simultaneously. The results showed that the WC of the brain diminished significantly in ischemic motor and somatosensory cortices. According to the statistical results, the signal amplitude, responsive area size, correlation, and wavelet coherence of the motor and the somatosensory cortices for stroke hemisphere were found to be significantly lower compared to the healthy hemisphere. The obtained results confirm that the OISi-based WC analysis is an efficient method to diagnose the relative severity of infarction and the size of the infarcted region after ischemic stroke.

## Introduction

Since the 1980s, optical intrinsic signal imaging (OISi) has been used as an optical imaging method in neuroscience. By measuring the reflected light from the cortex, one can measure the spontaneous and stimulus-evoked hemodynamic activities of the cortex^1^. This imaging method is based on capturing the minute reflectance changes from an illuminated brain, which occurs because of the changes in the blood flow, blood volume, and deoxygenated /oxygenated hemoglobin (Hbr / HbO2) concentration^1,2^.

One advantage of OISi is its capability in functional connectivity (FC) measurements of neocortex activities^3^. Up to now, many efforts have been made to measure the FC utilizing some imaging methods such as fMRI^4^, fNIRs^5^, and OISi^3,6^. In these imaging methods, the FC measurements are usually based on time or frequency evaluations^7–9^. In other words, the Pearson correlation metrics and coherence metrics are commonly used to measure FC in these imaging methods. These FC measurements are unable to identify the frequency dynamics and the time variations of the FCs, respectively^10^. Compared to other methods, OISi is very appropriate for the FC calculations due to its high spatial resolution, making it practical in visualizing the small functional domains of the cortex. Furthermore, this imaging method is capable to evaluate the hemodynamic changes in animal models of different neurological diseases such as epilepsy^11^, Alzheimer’s^6^, and ischemic stroke^3^.

In general, stroke is the most serious threat to the health, the fourth prominent reason for death, and the main reason for disability in the middle age and elderly people^12^. This neurologic disease not only causes death, but also incurs many mental and financial costs on the family of patients^12–14^. In 2014, OISi method was used to study ischemic stroke in mice^3^. In that study, the general differences were evaluated between the OISi signals and FC maps of a normal and a stroke group of mice. However, the differences were not assessed statistically. The FC of the cortex was evaluated in the time domain only and within the frequency ranges below 0.08 Hz. By using their results, the changes induced in high frequency hemodynamic oscillations such as cardiac, respiration, myogenic, and neurogenic activities after the stroke could not be recognized. In addition, their FC measurements were done in the time domain only.

The cerebral hemodynamic signals are composed of systemic activity and neurovascular coupling components^15^. The local cerebral metabolism is firmly coupled to local brain perfusion due to the anatomic and metabolic relationships between neurons, glial cells, and cortical arterioles which together comprise a neurovascular unit^16^. The main function of metabolic adjustment is to regulate the blood flow to satisfy the oxygen demand of cells^17^. One ability of the cerebral blood vessels is to maintain the cerebral blood flow (CBF) oscillations around a certain value. This occurs through myogenic, neurogenic, or metabolic mechanisms^18,19^. Myogenic mechanisms buffer minor variations in the CBF due to variations in systemic variables with the sympathetic nervous system being mostly activated when extensive pressure variations occur^20^. Changes of connectivity in myogenic range mainly reflects altered neurovascular coupling levels or myogenic activity^21^.

**Figure 1:**
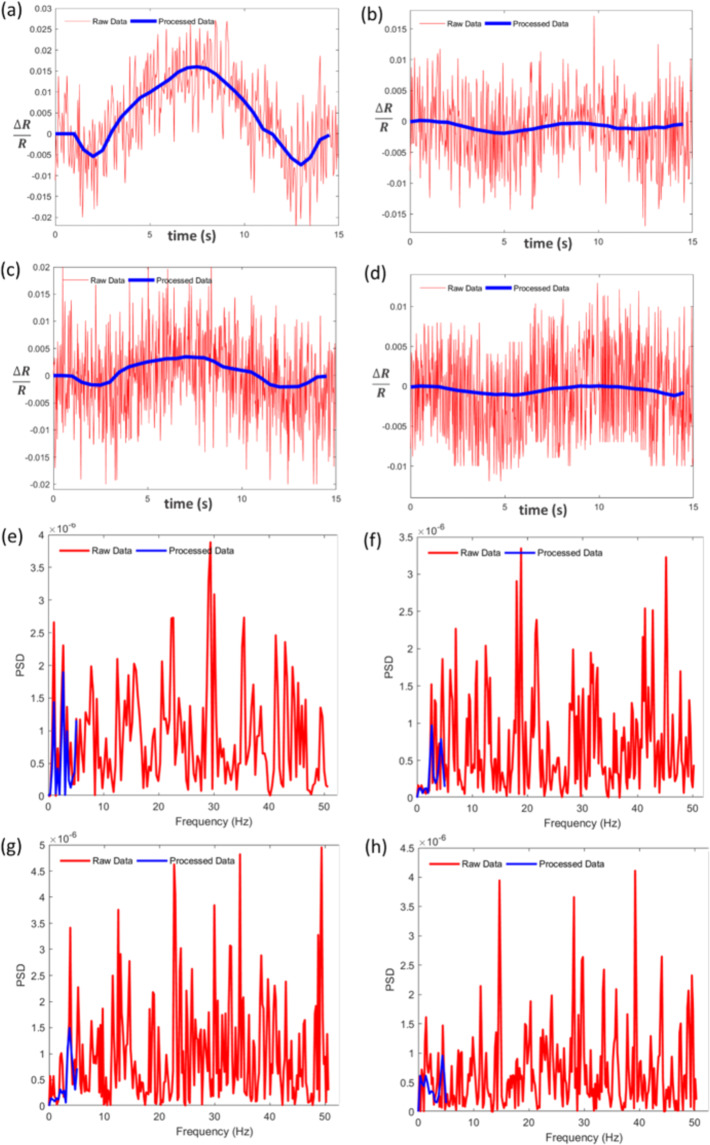
(**a**,**d**) Hemodynamic intrinsic signal evoked by whisker stimulation for (**a**) healthy motor, (**b**) stroke motor, (**c**) healthy somatosensory, and (**d**) stroke somatosensory cortices. The signals for raw data and signals after pre-processing analysis are shown in red and blue colors, respectively. e-h. PSD for (**e**) healthy motor, (**f**) stroke motor, (**g**) healthy somatosensory, and (**h**) stroke somatosensory cortices, related to (**a**–**d**) panels, respectively. PSD for raw data and data after pre-processing analysis are shown in red and blue colors, respectively.

In the current study, we used the wavelet coherence (WC) analysis to extract more features of the FC of the cortex. The WC shows the local correlation between the two time-series in the time-frequency domain^22,23^. Indeed, the simultaneous operation of two regions would lead to high value of WC due to formation of FC between these two regions. One advantage of the WC approach is its ability to investigate the dynamic features of the FCs in both time and frequency domains and the dynamics of phase relations in the FCs. In this paper, the dynamical characteristics of the FCs of the motor and somatosensory cortices were investigated in stroke-suffering rats using the WC analysis. In the literature, the FCs of the OISi data were calculated in frequencies ranging from 0.009 to 0.08 Hz^3,24,25^. In these studies, the FCs of the OISi time series only used correlation metrics to measure the FCs and thus did not have the capability to assess the FC dynamics. In this paper, using the WC analysis, the dynamic characteristics of the FCs obtained from the OISi time series were investigated in time and frequency domains simultaneously. In this work, by selecting a wide frequency range based on the power spectral density (PSD) of the obtained time series (Fig. 1(e–h)), one could measure the FCs in a broader frequency range than the 0.009–0.08 Hz range to assess the FCs originating from several sources of hemodynamic events. Utilizing the new approach proposed in this study, the OISi data were used to evaluate the changes induced in neurogenic, myogenic, cardiac, and respiratory oscillations after ischemic stroke in rats. These factors may indicate the systemic regulation activities and neurovascular coupling.

Adding the wavelet transformation to the OISi technique can improve the diagnosis of stroke severity and the size of infarcted region after ischemic stroke induction in experimental animals. In conventional methods used to measure the FC such as correlation- and coherence-based methods, it is not possible to determine the direction of the connections and the contribution of each of the underlying sources to the hemodynamic response and the temporal variations of the obtained connections^10^. On the other hand, due to its nature, the hemodynamic response is non-stationary and contaminated with different physiological sources and even noise. Hence, the authors were motivated to use the WC analysis to obtain the frequency-specific features of the FC, the direction of the connections by evaluating the changes in phase relationships over time and across frequencies, and the temporal variations of the FC.

The wavelet transformation has a variable time and frequency resolution throughout the time-frequency domain. Hence, it can provide a good temporal resolution at high frequencies and a good frequency resolution at low frequencies. Therefore, it is very useful for extracting frequency- and time-varying information from time series simultaneously. In general, this transform is more suitable than the other methods to separate intermittent, transient, and aperiodic signal components. It is therefore more suitable than the other methods to analyze biological signals^26^.

Using the wavelet analysis, the phase lag and coherence between two time series can be investigated as a function of both time and frequency. Accordingly, the proposed framework in this study is well-suited to study the nonstationary variations in the coupling between the time series extracted from the OISi. It can also examine the FCs that emerge as a result of stimulus and their changes over time.

In this work, the WC analysis advantages were used to study the intrinsic signal dynamics of stroke-suffering rats. The intrinsic signal of a cortex after stroke may have an abnormal behavior. The abnormality of the intrinsic signal due to stroke could be measured by the changes in the amplitude, latency, and size of the activated area. However, the changes in the intrinsic signal after stroke might also be due to impairments within the vascular system and perfusion which are difficult to detect. Furthermore, the likely changes in the FC due to the variations in the temporal relations among the brain regions might be used to detect the abnormalities after stroke. Thus, using traditional FC measurements which cannot assess temporal dynamics is not appropriate. By inherently assessing the temporal and frequency-specific information, the WC analysis can properly detect the abnormal responses by investigating the changes of the FC. Therefore, the WC analysis is a suitable measure for investigating the behavior of the intrinsic signals after stroke. The WC analysis is a useful tool for analyzing time series in both time and frequency domains. In particular, the temporal changes of the phase shift between the time series in different ranges of frequencies can be computed.

In conclusion, the WC analysis is an efficient method to obtain the time, frequency, and phase dynamics of the FC comprehensively and simultaneously with an appropriate resolution. This method of analysis is widely applicable to healthy and diseased brains. By measuring the WC (which is a measure of FC in the time-frequency domain) and comparing it between the healthy and stroke hemispheres at different time-frequency intervals, the relative severity of the damage resulting from the stroke can be measured. By using statistical analyses (especially the two-way ANOVA), the size of the areas with a significant difference in the WC can be used to determine the size of the affected area more precisely.

## Results

### The effect of cerebral ischemia on the scores of neurological deficits

The accuracy of the ischemic stroke induction in rats was checked through behavioral assessment. After this evaluation, it was realized that the limbs of the opposite side of the MCAO group did not respond to the stimuli. Further, the animal could not keep their balance on the smooth surface and instead of walking along a straight path, they moved in a circular path and could not climb the ramp. So, it was concluded that the stroke was induced. All these suggest that the ischemic rats endured the significant neurological deficits in comparison with the control group (Supplementary Table S2).

### TTC staining results

After imaging and TTC staining, the unstained (white) areas of the brain were defined as infarcted tissue. The stained (red) zones were considered as normal tissue. The infarcted area is the necrotic tissue suffering from neuronal death due to the lack of adequate blood supply. The images of the brain slices after TTC staining are shown in Supplementary Fig. S2b. The infarction volumes in the MCAO group of rats using TTC staining are also shown in supplementary Table S8. By using Paxinos atlas of the rat brain^27^, it was shown that the primary motor cortex (M1) and the primary somatosensory cortex jaw region (S1J) had been damaged (as illustrated in the second brain slice of Supplementary Fig. S2b). Furthermore, infarction was developed in the primary somatosensory cortex forelimb region (S1FL), the primary somatosensory cortex dysgranular region (S1DZ), and the primary somatosensory cortex, upper lip region (S1ULp) (as manifested in the third brain slice). By matching the fourth brain slice with the Paxinos atlas, the extension of infarction was observed in the primary somatosensory cortex hindlimb region (S1HL), the primary somatosensory cortex barrel field (S1BF), and the secondary somatosensory cortex (S2). In the fifth brain slice, the existence of infarction in the secondary auditory cortex (Au) and the secondary visual cortex (V2; in some rats) was observed. According to the mentioned data, the neurological deficit in the behavioral tests is justifiable.

### Differences between the intrinsic signal characteristics of healthy and ischemic hemispheres

Before comparing the intrinsic signals of healthy and stroke hemispheres, it is required to enhance the SNR of the time series for a more accurate comparison. The red-colored curves in Fig. 1(a–d) show that the stimulation-evoked hemodynamic changes are contaminated by different types of noises which will finally lead to wrong conclusions. Hence, different analyzing algorithms were applied to raw experimental time series to reduce the noises and enhance the SNRs. After analyzing the time series by registering the images, applying the Gaussian filter, and temporal averaging, the various phases of the evoked intrinsic signals due to the hemodynamic changes of the brain were mapped with a better SNR (the blue-colored curves in Fig. 1(a–d)). As can be seen in the PSD plots (Fig. 1(e–h)), the OISi data have several frequencies including 0.12, 0.34, 1.67, and 4.35 Hz, which can probably be attributed to myogenic, neurogenic, respiratory, and cardiac activities, respectively. In addition, in the OISi data, several frequencies in the range of 5 Hz and higher are also observed (the red-colored curves in Fig. 1(e–h)), which may be related to the systematic noises. Using the Gaussian filter, image registration, and temporal averaging, the high frequency noises were eliminated in accordance with the Nyquist limit in the new time series. The PSD at low frequencies is also partially attenuated compared to that of raw data (the blue-colored curves in Fig. 1(e–h)). These attenuations likely contributed to the enhancement of the SNR.

**Figure 2:**
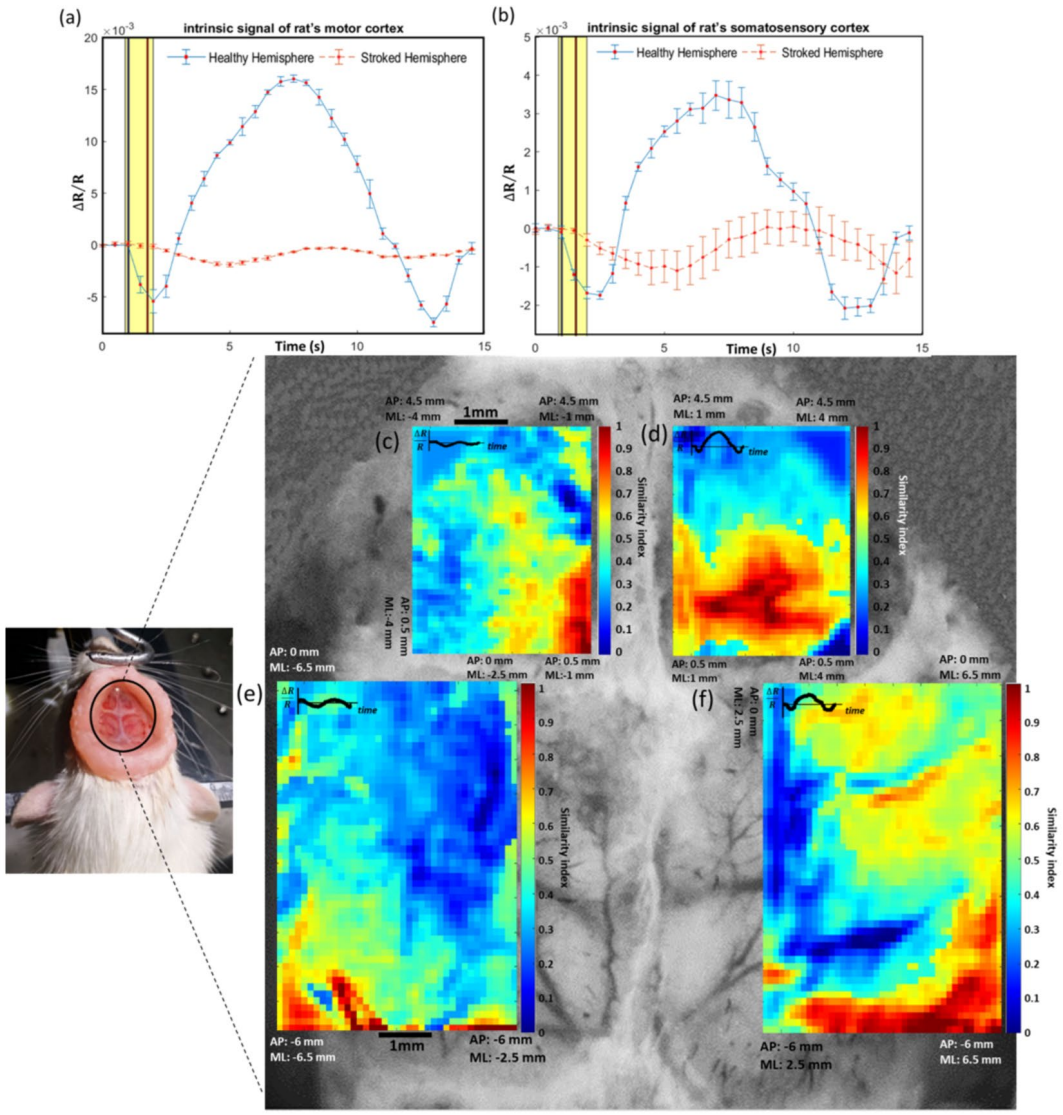
(**a**,**b**) The comparison of the signal amplitude and the latency of the intrinsic signals evoked by stimulating contralateral whiskers in the ischemic and healthy hemispheres. The yellow rectangle and colored bars show the stimulation period and the latency of the responses, respectively. The latency of signal in healthy and stroke hemisphere are shown in blue and red bars, respectively. The comparison between the spatial extent of the intrinsic response evoked by stimulating contralateral whiskers is shown in the motor (**c**,**d**) and somatosensory (**e**,**f**) cortices. c and e are related to the stroke hemisphere and (**d**,**f**) are related to the healthy hemisphere. The regions in red color illustrate the areas activated in response to stimulation. The calculations are done for every 10 × 10-pixel block and the coordinates of the brain areas under study and the spatial scale are shown in this figure. The waveforms of the expected responses used in the ‘pattern detection algorithm’ in the panels (**c**–**f**) are shown in the upper left corners of these panels.

To evaluate the changes induced by stroke, we compared the amplitude of the intrinsic signals evoked by whisker stimulation in the damaged and healthy hemispheres. Some studies^28,29^ have shown that after a stroke, the sensory system is impaired due to motor impairment^28^. On the other hand, whiskers are the most important sensory organs in rats and play an important role in many of their behaviors and functions^30^. Considering many factors such as the high sensitivity of rats to whisker stimulation, the easy accessibility of the whiskers, the rats’ quick exhibition of emotional or behavioral responses to whisker stimulation^31^, and the high coupling between the activity of the sensory neurons and the forces acting on the whiskers in both anesthetized and awake rats^30,32^, we found this type of stimulation appropriate for measuring the changes induced in the sensory function after stroke in the rats’ cortex. On the other hand, both the primary motor cortex and the primary somatosensory cortex are activated by whisker stimulation^33–35^. As a result, by stimulating the rats’ whiskers, one can investigate the effects of stroke in a larger area of the cortex. It was demonstrated that after the stroke, the amplitude of the responses to the whisker stimulations diminished remarkably in the affected hemisphere. We then compared the temporal features of the time-series in the damaged and healthy regions of the cortex. The results revealed a delay between the infarcted and the non-infarcted regions (Fig. 2a,b) i.e. the latency of the intrinsic signals shifted to higher values in the affected hemisphere. The independent sample t-test analysis showed that this shift is significant (Supplementary Table S3).

By implementing the signal similarity analysis on the obtained data, we also observed that the size of the region responding to the stimulation within the somatosensory and motor cortices shrank in the ischemic hemisphere, corresponding to the induced infarct (Fig. 2c–f). The regions responding to stimulation within both cortices are easily distinguishable in Supplementary Figure S3 by thresholding the images related to Fig. 2(c–f) to better highlight the activated regions.

Next, we evaluated the FC of the cortex by calculating the Pearson correlation between every 10 × 10-pixel block. Then, the average values of the Pearson correlation coefficients over all block pairs were considered as the FC strengths^36^. By comparing the FC strengths in healthy and stroke hemispheres, we found that the FC was reduced in the somatosensory and the motor cortices in the infarcted hemisphere relative to the healthy hemisphere (Supplementary Table S3).

### Wavelet coherence analysis

There are many cerebral oscillations in frequency ranges (except for those below 0.08) that affect hemodynamic fluctuations. By using the WC analysis, the FCs of the somatosensory and motor cortices were calculated in the frequency range up to 4.5 Hz. This upper bound frequency was selected to include the heart rate frequencies in the calculations (according to Fig. 1(e–h)). In the current work, the frequency-specific FC was assessed in the motor and somatosensory cortices in the two groups using the wavelet-based coherence analysis. Furthermore, by using this method, FC was calculated in time and frequency domains simultaneously.

**Figure 3:**
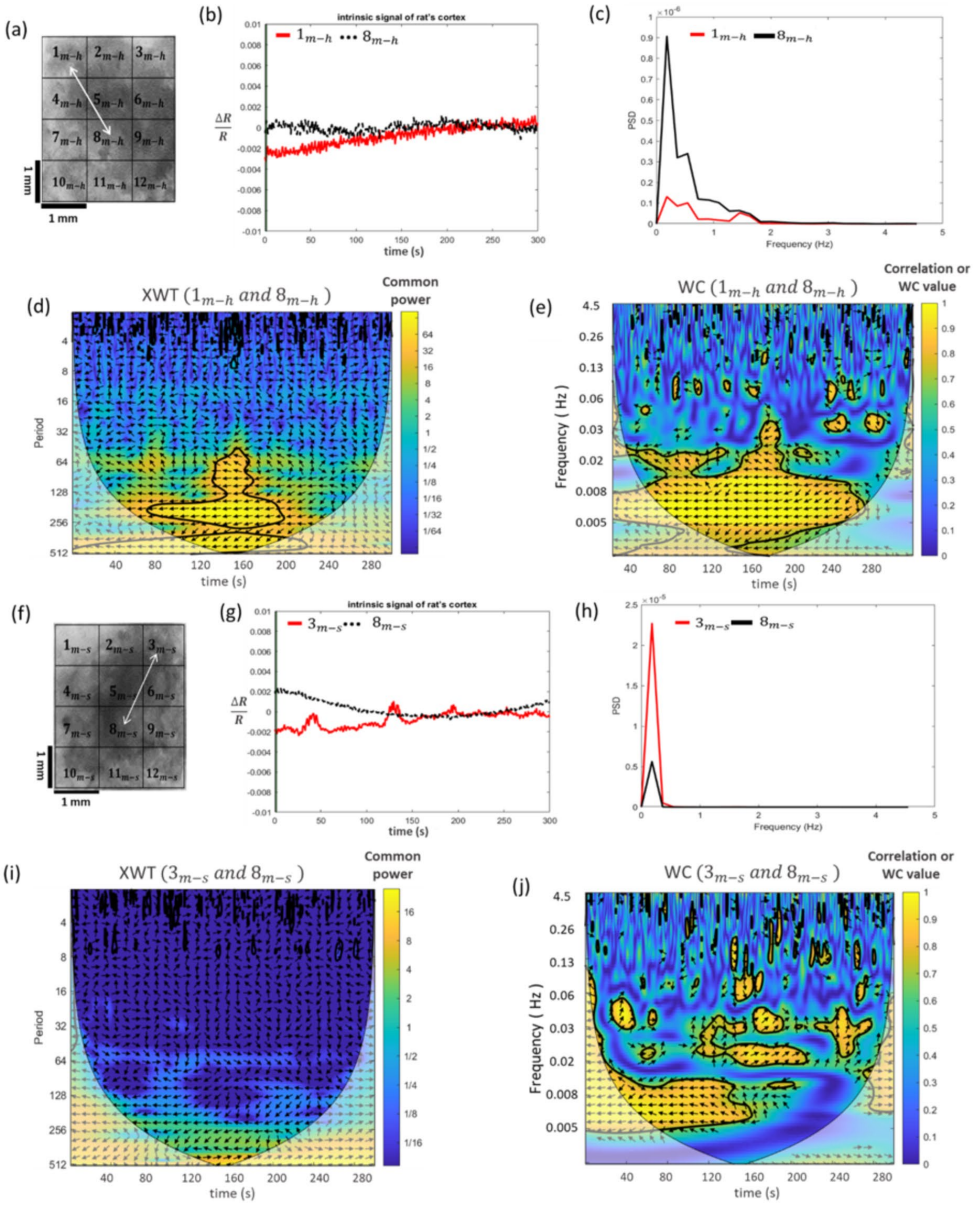
The comparison of the XWT and WC maps of the healthy and ischemic motor cortices. (**a**) Healthy and (**f**) ischemic motor cortices. Two randomly selected blocks in both hemispheres are displayed by white arrows. (**b**,**g**) are the time series of each selected block and are related to (**a**) and (**f**), respectively. c and h are PSD of each selected block and are related to a and f, respectively. (**d**,**i**) are the XWT maps of the selected blocks in healthy and ischemic motor cortices, respectively. The relative phase between two time-series is displayed by arrows in these plots. (**e**,**j**) are the WC maps of the selected blocks in healthy and ischemic motor cortices, respectively. The color bars in WC maps show the WC power values that indicate the areas in the time-frequency domain where two time-series co-vary.

By using the WC transformation, the coherence structure between the intrinsic hemodynamic signals was calculated. To calculate the WCs, each of the somatosensory and motor cortices for both the healthy and stroke hemispheres was divided into 12 sub-regions (blocks) (Supplementary Fig. S2a). Then, the CWTs of the time-series related to each block were calculated. Finally, the XWT and WC between all pairs of blocks were calculated separately. In order to compare the results of the two groups, a pair of blocks in the healthy motor cortex was randomly selected and a pair of blocks corresponding to these selected blocks in the stroke hemisphere was also chosen. A visual instance of this comparison has been shown in Fig. 3.

**Figure 4:**
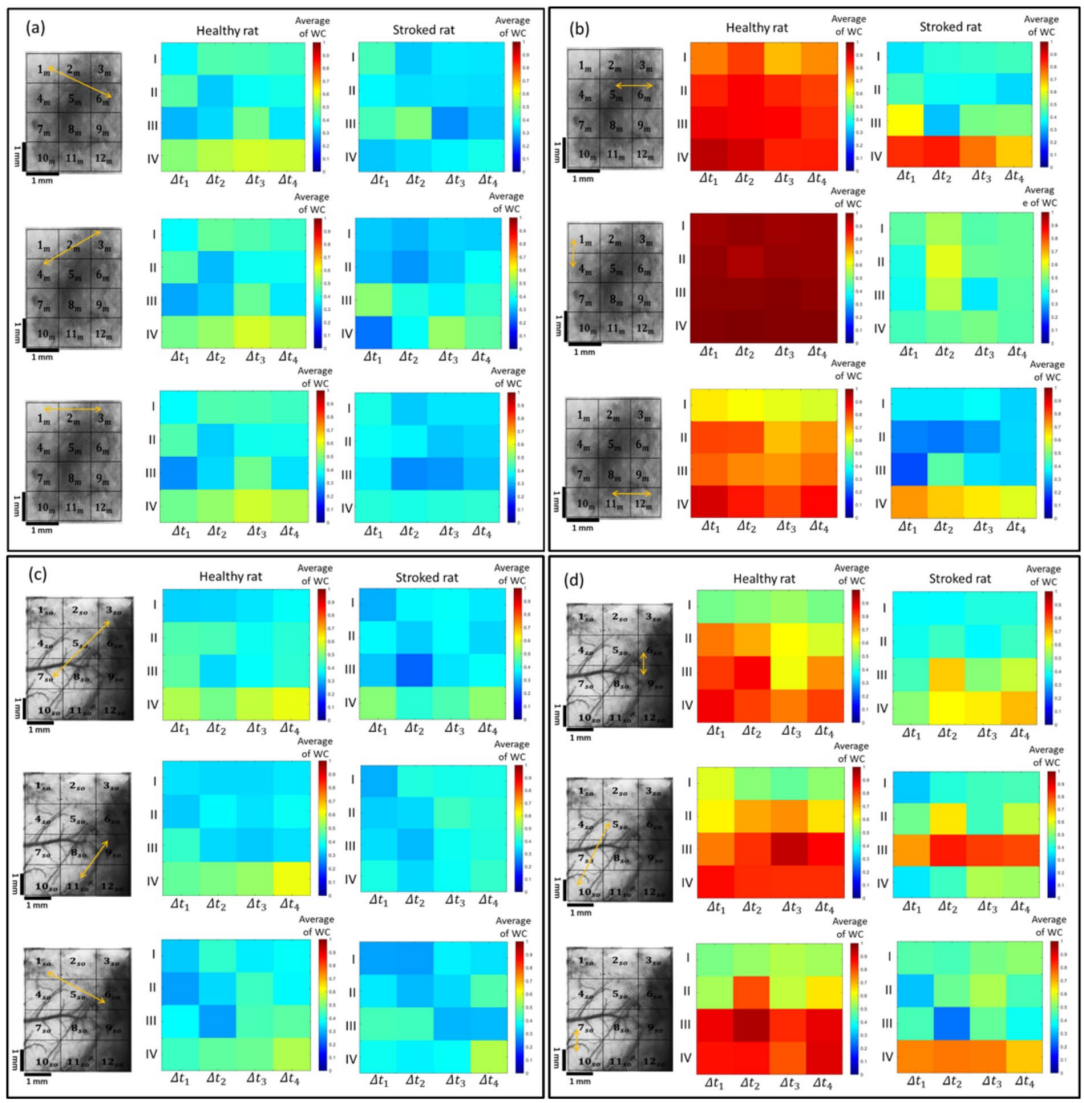
The averaged WC values in the 16 time-frequency domains in the motor (**a**,**b**) and somatosensory (**c**,**d**) cortices. Figures (**a,c**) illustrate relatively similar maps whereas Figures (**b,d**) illustrate different maps. The blocks are selected symmetrically in the healthy and stroke hemispheres.

The wavelet coherences are shown by two colored WC plots (Fig. 3(e,j)) in which the warmer colors (closer to yellow) represent higher local correlations in the time-frequency domain. The relevance of the colors and the correlation values are shown by the color scale bars. We were able to use these plots to investigate the WC variations of the selected blocks across both time and frequency domains. The regions enclosed by thick black curves represent the areas with significant correlations as estimated by Monte Carlo simulations. In the XWT plots (Fig. 3(d,i)), the regions with high common power are also illustrated with yellow color. We were able to utilize these plots to assess the variations of the common power between the time series of the selected blocks over the time and frequency domains. The thin black line or the cone of influence arising from the boundary conditions separates the areas with non-valid and valid estimations by shade and bright colors, respectively. The relative phase between the two time-series are shown by the arrows on these plots. Right-oriented arrows indicate the in-phase status of two time-series (or positive correlation), while the left-oriented arrows indicate the anti-phase status (or negative correlation). The downward arrows show the leadership of the first time series by 90 degrees and the upward arrows indicate the delay of the first time series by 90 degrees. These latter two conditions hold for the in-phase state. However, for the anti-phase state, they inversely hold. In the XWT plot of the stroke hemisphere in Fig. 3(i), it can be observed that for the two randomly selected blocks, there is no significant change in the common power between the two time series over the time and frequency domains and the phase direction is almost random everywhere. However, in the area enclosed within the black curve in the XWT plot of the healthy hemisphere (Fig. 3(d)), two time series have a high common power. Furthermore, the phase direction between the two time series is random in almost all the areas except for parts of the high common power region. The results of the WC plots also show that for the blocks selected in the healthy hemisphere over a larger area of time and frequency, two time series are correlated and this correlation is statistically significant. The arrow directions in this area show that the time series are in the antiphase state and have a negative correlation (Fig. 3(e)). However, in the WC plots of the stroke hemisphere, there are smaller correlation areas than those of the healthy hemisphere. In most correlated regions, there is a delay between the two time series. Due to the random arrows, the leader changes in all areas (Fig. 3(j)). Given these results, comparing the WCs of the two groups (the stroke and healthy hemispheres) is a bit difficult and time-consuming. For an easier and more precise comparison, based on the physiological origins of the cortex hemodynamic fluctuations, the frequency range was divided into four intervals and the average WC was calculated in these intervals. These intervals include I (2–4.5 Hz), II (0.4–2 Hz), III (0.15–0.4 Hz), and IV (0.05–0.15 Hz) which correspond to cardiac, respiratory, myogenic, and neurogenic activities, respectively^37^. To compare the stimulus-related WCs, the time domain was also divided into four temporal intervals including 1 s before stimulation (Δt_1_), 1 s during stimulation (Δt_2_), the first (Δt_3_), and the second 6.5 s after stimulation (Δt_4_). The average WC values in the specific time-frequency domain were obtained by the averaging method. The coherence values in I and II ranges indicate the synchrony of cardiac and respiratory activities within the cortex. The results of the current study suggest that stroke causes a disruption in stimulus-related average WC in all the specified frequencies. Furthermore, it was found that the average WC value in the frequency range I was significantly higher than those of the other ranges in both groups. To have a comparative view, a few examples of the average WC maps in both healthy and stroke motor cortices are shown in Fig. 4. As can be inferred from this figure, there are some regions of the cortex where the average WC values do not differ significantly in healthy and stroke hemispheres (Fig. 4a,c). However, in some other regions, there is a significant difference between the two hemispheres (Fig. 4b,d).

**Figure 5:**
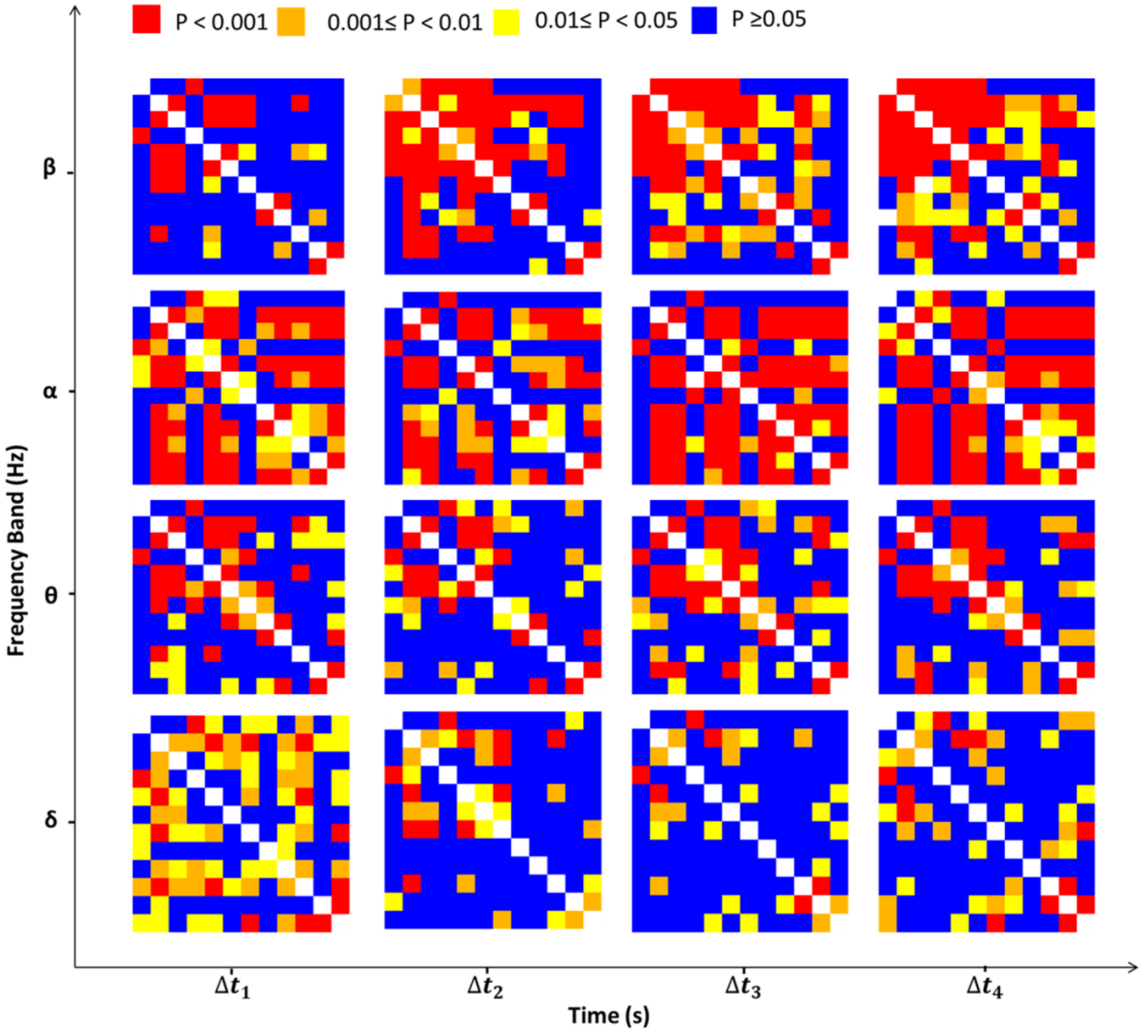
The statistical matrixes for the 16 time-frequency domains. The elements of the matrixes indicate the P-values of the two-way ANOVA analyses between stimulus-related WCs in healthy and stroke hemispheres. The red, orange, yellow, blue, and white colors show the P-values of <0.001, <0.01, <0.05, ≥0.05, and 0, respectively.

To examine the differences in the wavelet-based FC accurately, to investigate the changes induced in the wavelet-based FC in specific frequencies, and to evaluate the size of the affected area of the cortex, the differences in the average WC values of each pair of blocks (as shown in Supplementary Fig. S2a) within the healthy cortex in comparing to the corresponding pair of blocks within the stroke cortex were statistically assessed within the motor and somatosensory cortices. Afterward, the two-way ANOVA analysis was used for investigating the differences between the two groups regarding all the 16 time-frequency domains for all pairs of blocks. The results for the motor cortex are shown in Fig. 5. The statistical analysis resulted in 16 statistical matrixes. Any statistical matrix has 12 × 12 components each of which indicates the value of the statistical difference (in terms of P-value) in the average WC value of a certain pair of blocks between the healthy and stroke hemispheres. The diagonal elements of the statistical matrixes represent the differences between the average WC values of each block and itself and are equal to zero. The significant differences between the pairs of blocks with the P-values of <0.001, <0.01, <0.05 are shown in red, orange, and yellow colors, respectively, while the no significant differences with the P-values of ≥0.05 are shown in blue color. The diagonal elements corresponding to zero are shown in white color. The statistical results related to these statistical matrixes demonstrated that there are significant differences between the healthy and stroke hemispheres in range II in all time intervals and in frequency range I during the time intervals of Δt_2_, Δt_3_, and Δt_4_ in most pairs of blocks. There is also a significant difference between the two groups in the frequency range IV in the time interval of ∆*t*_1_.

**Figure 6:**
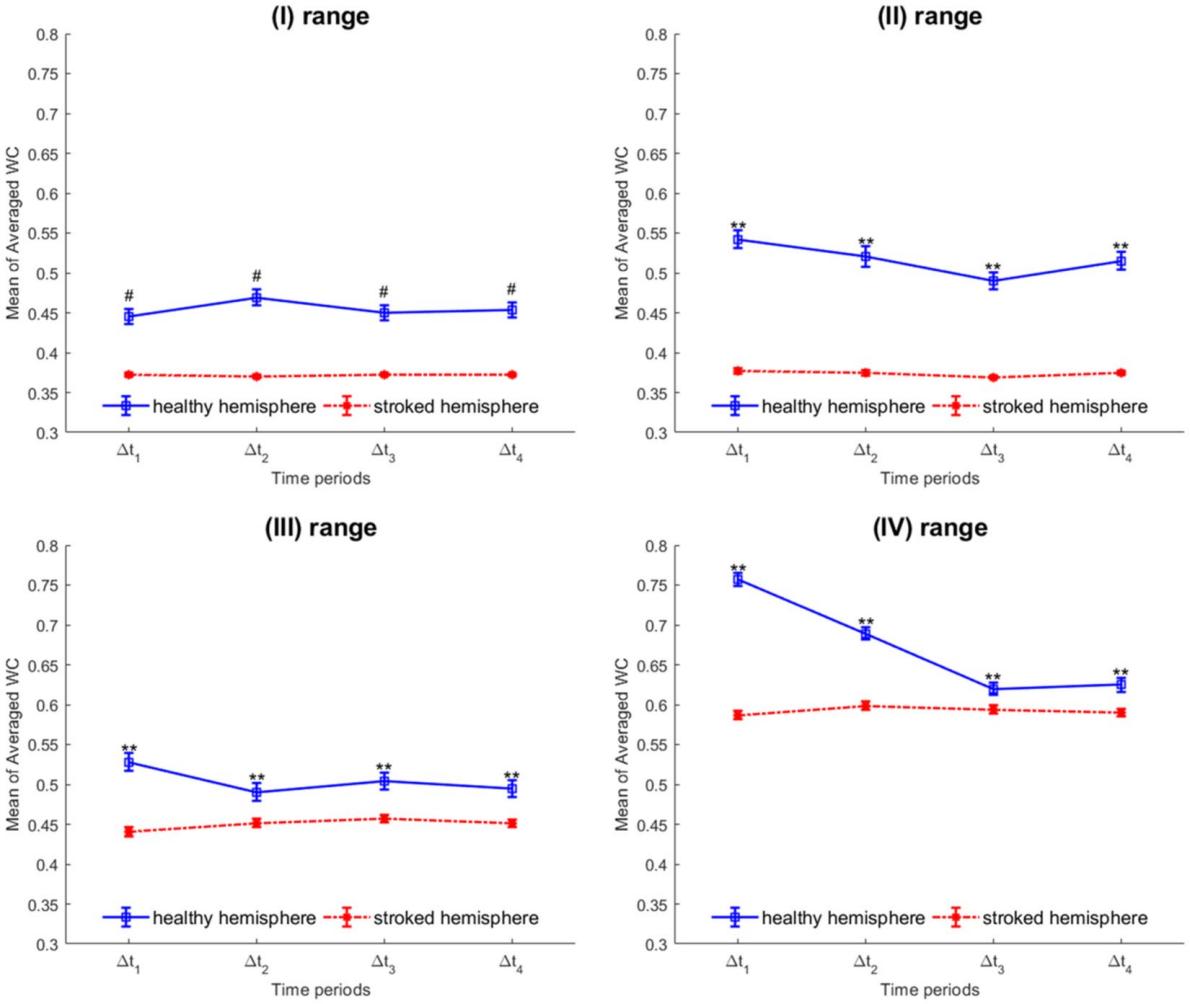
The mean WC value for the 16 time-frequency domains in the healthy and ischemic hemispheres averaged across all blocks within the motor cortex. The mean and the standard error of the mean of the average WC values at all time-frequency domains are illustrated in this figure. The P value of the ANOVA analysis is written on each related graph. **P < 0.01; ^#^P < 0.00001.

For each specified time-frequency domain, the average WC values of all pairs of blocks were averaged and then the results between the two hemispheres were compared. The statistical differences between the two groups were calculated by employing the one-way ANOVA analysis. The mean and the standard error of mean of the average WC in each frequency range for all time intervals in the motor cortex are displayed in Fig. 6. As can be seen in this figure, in all the 16 time-frequency domains, there are significant differences between the two groups. Furthermore, the reduction in the average WC value in the stroke hemispheres in the 16 time-frequency domains and in all specified frequency ranges can be observed in this figure.

**Figure 7:**
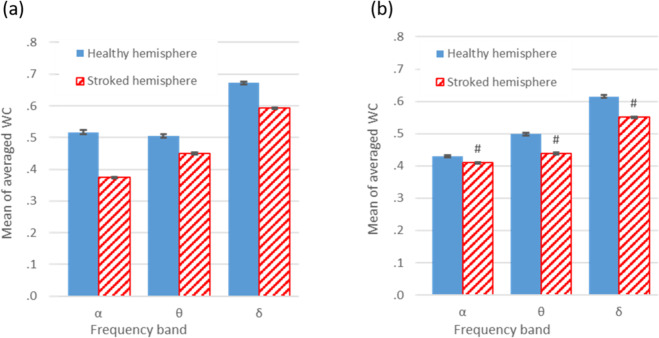
The comparison of the average WC values in the specified frequencies between the healthy and stroke hemispheres in (**a**) the motor cortex and in (**b**) the somatosensory cortex. The P value of the ANOVA analysis is written on each related graph. *P < 0.05; **P < 0.01; ***P < 0.001; ****P < 0.0001; ^#^P < 0.00001; n.s. P ≥ 0.05.

To calculate the average WC in the specified frequency ranges, the average WC value of each of the 16 time-frequency domains was averaged across all time intervals. The mean of the average WC value in each frequency range of the motor and somatosensory cortices is displayed in Fig. 7.

The results show that in both cortices and in all frequency domains, a decrease in the value of the average WC occurred in the stroke hemisphere. Finally, the mean of the average WC value was computed across all time-frequency domains. Then this value was compared between the healthy and stroke hemispheres in both the motor and somatosensory cortices. Using the independent sample t-test analysis, a significant difference between the two groups in both cortices was observed.

### Numerical and statistical analyses

The significance of the differences between the control and stroke groups was measured using the t-test and ANOVA. To ensure that the assumptions required for parameter analysis were fulfilled, the normality and variance homogeneity of the data of each group were tested using the Kolmogorov–Smirnov test and the Levene test, respectively. Bonferroni comparison tests were used for the post-hoc analyses of the two groups. The numerical and statistical results were reported in detail in the Supplementary Information file.

## Discussion

In the current work, a new approach was used to measure the FC of the cortex based on the OISi data after ischemic stroke induction in rats. This method allows the variations of the FC to be assessed simultaneously in the time and frequency domains. Furthermore, the dynamic features of the phase relationships in FC measurements can be investigated by using this approach. The current OISi-based FC methods primarily measure the FC using correlation metrics and cannot investigate the FC dynamics. In addition, there have not been studies so far on changes in FC based OISi data at frequencies other than 0.009–0.08 Hz following stroke. In contrast, by using the proposed approach, OISi becomes a useful method for measuring the FC of the cortex in the time-frequency domains simultaneously. In this study, the features of time variations as well as the phase and frequency dynamics of the FC were measured in a wider frequency range than 0.009–0.08 Hz. Because, in the wavelet-based FC in the OISi method, the focus is on measuring the connections between the brain regions in the time and frequency domains simultaneously. Furthermore, in this study, the FCs of the cortex were examined based on the OISi data in the wide frequency range of up to 4.5 Hz which includes hemodynamic activities such as neurogenic, myogenic, respiratory, and cardiac oscillations in the cortex. In this study, these activities considered in correspond to I, II, III, and IV frequency ranges, respectively. The upper bound frequency in the wavelet analysis was considered to be 4.5 Hz (according to frequency distribution of our OISi data as can be seen in Fig. 1(e–h)) to include the heart rate frequencies in our investigations^37^. It can be inferred from the results that the WC and therefore the FC of the motor and somatosensory cortices were reduced remarkably in all time and frequency intervals after stroke.

Moreover, in this study, the characteristics of the intrinsic signal and FC in healthy and stroke hemispheres of rats were numerically compared for the first time. In the current work, the motor and somatosensory cortices of the rats were imaged by stimulating their whiskers for 1 s at 5 Hz. At first, the amplitude and time features of the intrinsic signal in both hemispheres of the control and stroke rats were measured by stimulating contralateral whiskers. After comparing the two hemispheres in the control rats, it was concluded that there is no significant difference in the mentioned parameters as reported previously^3^. However, in the stroke group, the comparison results showed that the intrinsic signal amplitude was significantly reduced after stroke. Furthermore, the temporal characteristics of the signal indicated that the signal latency has significantly increased in the stroke cortices compared to the healthy ones (Supplementary Table S3 and Fig. 2). It was also found that the size of the activated regions of the cortex in response to stimulation was remarkably reduced due to stroke (Supplementary Table S3 and Fig. 2). This is in accordance with studies on brain responses measured in forelimb stimulation in rats^38^ and mice^3^. It should be also noted that in this study, it was attempted to use such comprehensive tests as limb placing, symmetry of muscle tone, and sensory function which usually cover the sensory and motor behaviors of the animals. In cerebral ischemia induced by MCAO, the major injured cerebral regions are the cortex and striatum^39^. In addition, many of the neurological defects measured by behavioral tests are associated with damage to the sensory cortex, the motor cortex, and striatum areas following brain ischemia. Moreover, other behavioral tests including postural signs, gait disturbances, climbing, biased movement, and motility were used for assessing motor and sensory capabilities in control and MCAO groups. As can be seen in Supplementary Table S2, the total neurologic score of the MCAO group was 24.17. This score indicates that the motor and sensory regions of the cortex in the MCAO rats were damaged. After imaging, the rats of the MCAO group were sacrificed and TTC staining was performed on their brains. The reported results in section 3.2 and Supplementary Fig. S2b proved that some parts of the motor and somatosensory cortices were damaged after ischemia. It was hypothesized that the changes induced in the temporal features of the signal were in line with the damages induced in the motor and sensory capabilities mentioned in the behavioral test. The disruptions occurring in the signal amplitude and the size of the area responding to the whisker stimulation were also directly related to the infarcted regions of the motor and somatosensory cortices. The findings of the current study are consistent with those of previous studies on the changes occurring in the cortex after stroke in humans^40^ and animals^41,42^. Investigating the disruptions induced in the rats’ cortex can be helpful in understanding the pathophysiology of stroke in humans.

It is worth noting that OISi method was employed to assess the effects of the ischemic stroke on the FC of the motor and somatosensory cortices. During MCAO surgery, the MCA and ACA are occluded. As previously reported^43^, the dorsal branch of MCA directly supplies blood to the somatosensory cortex. In addition, the MCA and ACA are responsible for supplying blood to the motor cortex. Therefore, it is expected that the occlusion of these arteries results in tissue infarction in the motor and somatosensory cortices.

In line with other common methods for assessing the FC of the cortex, the Pearson correlation between the determined regions in the motor and somatosensory cortices was calculated. The means of the correlation values in the two hemispheres were compared in the two groups. The results showed that there was no significant difference in the resting-state and the stimulus-related FC of the motor and somatosensory cortices between the two hemispheres in the control group. However, the same comparison in the MCAO group revealed that FC was significantly weakened in the stroke hemisphere (Supplementary Table S3) which is in accordance with other studies conducted on rats and humans^44,45^. In other words, the correlation values were significantly lower in the infarcted regions. The lower correlation values in the stroke hemisphere in frequencies below 0.08 Hz suggests a disruption of neuronal connectivity networks due to stroke.

The disruptions in the motor and somatosensory FCs (Supplementary Table S3) are consistent with the damage induced in these cortices after ischemia as reported in a previous study^3^. The results showed that the lower correlation of the hemodynamic signals in frequencies below 0.08 Hz in infarcted regions reflected the lower neural connectivity between infarcted cortical areas. These FC disruptions are directly associated with the lesions resulting from artery occlusion in MCAO surgery.

In the current work, we mostly concentrated on the changes induced in the stimulus-related WC after stroke. The results showed a significant difference between the WC of the healthy and stroke states which are in line with infarctions induced after the ischemia (Fig. 7).

WC results showed that the hemodynamic oscillations reduced significantly in all specified frequency ranges after the stroke which may be interpreted as less synchronization of cardiac and respiration-based hemodynamic fluctuations and decline of the cerebral myogenic and neurogenic mechanisms in the stroke compared to the healthy hemisphere. These reductions mainly affected the larger area of both cortices in I and II frequency ranges (Fig. 5).

We found lowered WC value within I frequency range for the infarcted region compared to the healthy ones. The impaired synchronization in this range might signify that the blood flow distribution in the infarcted regions is affected by systemic factors. Naturally, the blood flow distribution can be controlled by the humoral and neural effect of the brain on the cardiovascular system^46^. In summary, the change in blood flow distribution might have significantly influenced the WC in the infarcted region in range I.

The reduced WC values within II frequency range in the motor and the somatosensory cortices may be reflecting the asynchrony of respiration effects inside these cerebral regions. These results can be in line with reduction of respiration rate in MCAO rats in other work^47^.

The significantly lower level of WC in III frequency range reflects the corresponding weakened synchronization of the myogenic activity or spontaneous contraction of the vascular smooth muscle cells. Indeed, the myogenic activity can influence the FC levels. On the other hand, the weakened WC and thus the reduced synchronization of the OISi signal in this frequency range means that stroke may impair the coupling of the nerve activity in the infarcted regions thus reducing the FC of the stroke hemisphere. Our results showed that the lower WC or FC levels within III frequency range were influenced by impaired myogenic mechanism and are in agreement with the results of^48^ in rats. Also, the impaired neurovascular coupling level may contribute to reducing the WC levels in this range.

The lower WC in IV frequency range reflects the asynchrony of the neurogenic activity in the infarcted region of the cortex. Thus, the reduction of the WC level in IV range indicated lowered FC strength which is associated with the neurological activity within cortical infarcted regions. In the present work, the infarcted hemisphere showed significant disruption in connectivity within the IV range. As in other studies^49,50^, it may be caused by impaired neurogenic activity after the stroke. These results reflected the weaker synchronization in cerebral connectivity due to induced disruption of vascular or neuronal factors after the stroke. Regarding the influence of the neurogenic activity on the vessel wall over hemodynamic oscillations within the IV range^51^, the lower FC in this range represents the relatively smaller contribution of neurogenic activity to the adjustment of blood perfusion of the brain in the infarcted hemisphere owing to stiffening of vessels. On the one hand, the disruption of the cerebral networks’ connectivity in the infarcted regions may indicate augmented vasculature stiffness. Meanwhile, in accordance with the literature, the vessel stiffness may lead to altered vessel walls and activation of many complex mechanisms involved in the atherosclerosis^52–54^.

Based on these comparisons, the experimental results of the current work provided greater insight into the changes induced by stroke in neural and metabolic communication within the motor and the somatosensory cortices. Therefore, by applying the wavelet analysis in OISi method, one could achieve an optimal tool for precisely evaluating the changes induced in FC after the ischemic stroke. It would be more interesting to record the electrophysiology data at the same time as recording the OISi data. In this case, using the information provided by the WC analysis, one would be able to obtain accurate information about the induced changes in the neuronal activity after the stroke.

In summary, the OISi method was improved by employing WC algorithm to analyze its data. The distinctive feature of this work is measuring the FC of the cortex simultaneously in the time and frequency domains and in frequencies up to 4.5 Hz, which are in the range of fast hemodynamic oscillations including cardiac, respiration, myogenic, and neurogenic activities. Utilizing the WC analysis, we could evaluate the differences induced in the frequency specific FC of the cortex after ischemia. WC was utilized in this study as a new FC measurement to characterize the changes occurring in the FC of the motor and somatosensory cortices in different frequency ranges after induction of ischemic stroke in rats. This information facilitates the diagnosis of stroke. It should be stated that the findings of the current study might lead to the possibility of using the wavelet transform of intrinsic signals in the assessment of cerebral atherosclerosis in subjects with a high-risk of stroke. The presence of vascular obstruction and therefore the probability of the risk of stroke can be evaluated by using the information provided by the current study. By employing this novel approach in the OISi method, one could detect not only the impaired functional connections of the cortex but also the existence of impaired myogenic and neurogenic activities caused by stroke. Therefore, it is expected that a decrease in these two activities could be an estimate of the impairment or defect in CA caused by stroke and infarction (in line with^55^ for rats) based on the contributions of the myogenic and neurogenic activities to CA^56–58^. It has been proved that CA is generally impaired after an ischemic stroke^58,59^. According to our findings, both myogenic and neurogenic activities were reduced or impaired after stroke. At first glance, it seems that these findings can be used to estimate the disruption of CA by considering the main contributions of myogenic and neurogenic activities to CA^56,58^. However, to assess the CA solely based on myogenic and neurogenic activities is challenging. We need to use a complementary method to measure ABP along with recording the hemodynamic activities (and thus measuring the CBF) so that the accuracy of the evaluations is increased and the assessment of the CA changes is validated. It should be noted that by recording the CBF and ABP together and utilizing the analysis method presented in this paper, one can measure the CA dynamics by measuring the WC between the ABP and CBF time series. Examining this factor by combining these two recordings is one of our suggestions for those interested in investigating the CA changes after neurovascular diseases such as stroke. Furthermore, based on the results of recent studies^29,60^, by measuring the coherence phase differences extracted from the WC analysis, the stiffness of arteries or atherosclerosis and consequently the risk of stroke could be assessed. This can be done by comparing the WC phases of healthy and infarcted regions of the brain. This is our suggestion to researchers interested in continuing our work. Based on the results, by relying on the inherent advantages of wavelet-based analysis including the ability to investigate the phase, time, and frequency dynamics of the FCs of the cortex simultaneously and by measuring these connections at higher frequencies related to fast hemodynamic fluctuations, the authors were able to propose an analysis algorithm for the OISi data which is efficient, fast, and accurate in the diagnosis of ischemic stroke and might be useful in neurological sciences.

Despite the advantages of the WC analysis, it has some complexities. The computational complexity of this study is due to the high volume of computations to extract multiple properties of the FC simultaneously. The WC analysis measures various factors such as time, frequency, and phase dynamic characteristics of the connections between two time series and also examines the statistical significance of the connections using the Monte Carlo method. Hence, the wavelet analysis is computationally heavy and time-consuming. Another complexity of the proposed method is the difficulty of interpreting the obtained results and comparing the WC and XWT plots between healthy and stroke hemispheres to find the differences between them. This difficulty arises from the fact that the number of the WC and XWT plots is high and that each plot contains several attributes. Furthermore, the current work has several limitations. One of them is due to the nature of hemodynamic time series. In other words, despite the ability of the WC to detect the changes in cortical FCs at different frequencies, since this imaging method merely measures the hemodynamic activities of the brain, discussing frequencies higher than that of cardiac activity (such as the frequency of brain waves) is not sufficiently valid and may involve systematic noises. To address this limitation, we suggest that the researchers interested in the OISi method integrate this imaging method with the LFP recording method. This allows them to discuss the FCs of the cortex in the brain wave frequencies and to investigate the neurovascular coupling. Another limitation of this work is its low SNR due to the presence of several noises including physiological noises, motion noises, and systematic noises during the imaging procedure. We managed to address this limitation using post-processing analyses mentioned in the Methods section. Finally, there were also several limitations during imaging such as animal breathing during imaging, post-stroke animal mortality due to severe brain injury, and the awakening of some animals during imaging (due to the anatomical and physiological differences of the animals in response to a specific dose of the anesthetic).

Our designed instrument has diagnostic aspects. We hope that this instrument and our proposed analysis protocol can be used as an intraoperative setup to help surgeons to identify the map of brain lesions, the obstruction of arteries, and the FC maps during surgery. Due to the high spatial resolution of the OISi method, the obtained FC maps using this method will have a high spatial precision. Furthermore, due to using the wavelet analysis in data processing, the extracted FC maps will have precise time, phase, and frequency dynamic features. The obtained information would be valuable during surgery.

## Methods

### Experimental setup

In the prepared setup, the cortex was illuminated by light emitting diodes (LEDs). To provide stabilized radiation, a large battery together with a 10,000 µF capacitor and voltage regulator were used to supply power for 6 parallel 1w power LEDs. The LED’s intensity fluctuations were stabilized to about 10^−4^–10^−5^. To have uniform illumination on the cortex, a circular holder was used containing 9 LED including 3 green LEDs and 6 red LEDs (with wavelength about 620–625 nm and with intensity about 50-60 lumen). The camera used for OISi setup was a 16 bit sCMOS with a well-capacity of about 30,000 electrons (Hamamatsu Orca Flash 4, v3), located vertically above the cortex. Also, an extension tube attached to a Nikon lens (Sigma 105 mm f/2.8 EX DG OS HSM Macro Lens) was applied to reduce the depth of field of the imaging system to about 300 µm, which is critical for reducing the surface vascularity artifacts in OISi^1^. For stimulating the rat’s whiskers, a metal rod attached to a stepper motor was used. Also, the head of the anesthetized rat was stabilized relative to the camera using a stereotaxic frame. Ultimately, the entire imaging setup was enclosed in a dark box. The schematic view and the assembled setup for OISi experiments are shown in Supplementary Fig. S1a,b respectively.

### Animal preparation

#### Group assignment

Twelve adult male Wistar rats (250–350 g) were housed under conditions of controlled temperature (22 ± 2 °C) and constant humidity with a 12-hour light/dark cycle. All the steps of preparing the animals and the imaging protocols have been based on the National Institute of Health and Guide for the Care and Use of Laboratory Animals (NIH Publications, revised in 2011). This study was approved by the Ethics Committee of Shahid Beheshti University of Iran. The rats were randomly divided into two main groups, each including six rats (control and MCAO). In both groups, the two hemispheres were compared to see the differences and then the results of the comparison in the control and MCAO rats were studied separately. The assessment of neurological defects and TTC were performed to ensure the induction of ischemia.

#### Induction of ischemic stroke in the rats

To induce ischemic stroke in the rats, middle cerebral artery occlusion (MCAO) surgery was performed. As a common method to induce focal ischemia in rats, MCAO surgery has many advantages including relatively fewer invasions, ease of use, and the possibility of reperfusion^61^.

For this purpose, the rats were intraperitoneally anesthetized with chloride hydrate (400 mg/kg) and were put in a backrest position on a surgical pad. A cut in the midline of the neck was created and then the tissues of the salivary glands were removed. Then the sternohyoid muscle was gently separated. The common carotid artery (CCA) is under this muscle. It was also carefully separated from the adjoining vagus nerve^61^. Afterward, the end part of the external carotid artery (ECA) and the first part of CCA were closed by a silk suture. At this stage of surgery, the internal carotid artery (ICA) was the only remaining extracranial branch of the CCA which supplied blood to the brain. Using the microvascular clip, the common point between CCA and ICA was held and then a hole between it and the closed point of the first part of the CCA was created with a pair of microsurgery scissors. Next, a 3-0 nylon intraluminal suture, that had been rounded by heating and coated with poly-l-lysine (Sigma, USA), was inserted into the hole and the microvascular clip was released. The nylon suture was softly conducted to the ICA until resistance to its passage was sensed. The area in which resistance to passage is sensed is, in fact, the location of the separation of the MCA from the Willis ring. The artery was blocked for 60 min using this nylon suture. Moreover, a silk suture was tied around the ICA and hence around the intraluminal nylon suture. After 60 minutes, the nylon suture was slowly withdrawn from the artery. Thereupon, the blood began flowing through the Willis ring. Finally, the surgical region was sewed^62^.

#### Behavioral assessment of the neurological defect caused by stroke

24 hours after reperfusion, a neurological evaluation was performed by behavioral assessment to check the accuracy of the ischemic stroke induction in MCAO group (n = 6). Moreover, this behavioral assessment was performed in the control group (n = 6) for comparison with the MCAO group. All of the tests were performed by a professional observer who was blind to the details of animal grouping. The behavioral assessment examined several factors such as postural signs (forelimb flexion and thorax rotation), biased movement to pulling the tail three times, biased movement to pushing back of body three times, limb placing (forelimb, hindlimb), symmetry of muscle tone and strength (lateral resistance, grasping strength), sensory function (grasping reflex of forepaw, touching reflex), gait disturbance, climbing, motility, and spontaneous activity (Supplementary Table S1). In this assessment, the rats’ sensory and motor capabilities were measured. To do this, the strength and balance of the rats’ organs were measured and their muscle symmetry was checked by assessing their ability to climb a ramp. In addition, the rats’ sensorimotor abilities in their organs such as ear and paw, the positioning of their limbs on a flat surface, the speed of their movement, and the symmetry of their organs during walking were also examined (Supplementary Table S1). The total neurological score was assigned as the sum of the partial scores (with a maximum point of 28 in the stroke rats and a minimum point of 0 in the normal rats)^63^. The results are shown in Supplementary Table S2. They were obtained using the evaluation of the scores of the Supplementary Table S1^63^ in the MCAO and control groups of rats.

#### Confirmation procedure of the induction of cerebral ischemia

To ensure the induction of ischemia, the triphenyl tetrazolium chloride (TTC) staining was performed after imaging. 26 hours after the ischemic surgery, the rats were killed after imaging and their brains were rapidly removed and cooled in saline water at 4 °C for 15 min. Coronal sections with the thickness of 2 mm were performed (Brain Matrix, Iran). The slices were immersed in a 2% 2,3,5-TTC solution (Merck, Germany) and kept in a water bath at 37 °C for 15 min^64^.

#### Animal preparation for imaging

To prepare the rats for imaging, they were first intraperitoneally anesthetized with urethane (1.25 g/kg). After removing the scalp, the rats’ skull on top of the motor and somatosensory cortices was thinned using a dental drill so much so that the cortical surface arteries were fully visible. To prevent skull overheating, the drilling was periodically stopped and an artificial cerebrospinal fluid (ACSF) was used to cool down the skull. Then, using a dental cement mixture, a wall was created around the thinned cranial region of the cortex and filled with ACSF or saline water to keep the surface arteries and the cortex clearly visible. Throughout the experiment, the animals’ temperature was kept at 37 °C using a thermal blanket. Then the exact position of the camera above the rats’ brain was set using an XYZ stage^2,65^.

#### Imaging protocol

First, a green light was illuminated on the rats’ skull and the cortex was imaged in focus. This image contains the cortical surface arteries and can be used as a reference image. Then the cortex was illuminated by a red light, the camera was focused about 400–600 µm below the surface of the cortex, and imaging was done at 100 fps. Defocusing the camera is crucial for minimizing the superficial blood vessel artifacts. The imaging trials in the current study included resting-state trials and stimulation trials. The resting-state trials included imaging the cortex for about 300 s without applying any stimulation. In contrast, the stimulation trials included 315 s imaging of the cortex along with applying stimulation. The stimulation trials included 9 stimulation epochs with one control epoch included between every two consecutive stimulation epochs. Every stimulation epoch comprised 1 s resting-state imaging, 1 s imaging during the whisker stimulation, and 13 s resting-state imaging after stimulation. The control epochs included 21 s resting-state imaging. Therefore, the resting interval between consecutive stimulus deliveries was about 34 s. Each trial was repeated four times. To reduce the image shot noise, a 2 × 2-pixel binning was applied. The stimulation was done by the deflection of C1-3 and D1-3 whiskers about 0.8 mm in rostral-caudal fashion at 5 Hz.

In this study, the motor (Bregma, 0.5 to 4.5 mm; Lateral, 1 to 4 mm) and somatosensory cortices (Bregma, 0 to −6 mm; Lateral, 2.5 to 6.5 mm) in both hemispheres of the healthy and stroke rats were imaged according to the Paxinos atlas of the rat brain^27^.

### The analysis protocol

#### Calculating the time series of the intrinsic signals

The experimental procedure was controlled by LABVIEW software interface and the data were analyzed by MATLAB software. In analyzing procedure, at first, the noises due to motion were reduced using intensity-based digital image registration condition^66–68^. The image registration algorithm was applied for registering all the acquired images before analyzing them. For this purpose, the images were registered using an intensity-based optimization. In this step, a similarity metric was used iteratively as the optimizer input to compensate the displacement and rotational changes of the images^69^. The image registration algorithm managed to correct almost all the displacements and rotations induced in the images during the experiment.

Afterward, the fractional value (FV) of the reflected light from the cortex was calculated^2,70^ on a pixel-by-pixel basis relative to the average intensity of all frames before the stimulus onset. This average intensity is called R_B_ (baseline reflection) and the reflection in every frame is named as R_i_ (for i = 1… n, where n is the total number of frames taken):

1 $$FV=\frac{\Delta R}{R}=\frac{{R}_{i}-{R}_{B}}{{R}_{B}}$$

Then, a Gaussian filter with a kernel size of 5 pixels was applied to obtain smoother images and to eliminate the high spatial frequency noise that was probably due to the shot noise. Next, to increase the signal to noise ratio (SNR), the temporal simple averaging of the images was used (the time window size is selected to be about 10 time points). Utilizing this procedure, temporal downsampling was performed by simply averaging over consecutive frames and the temporal resolution decreased from 100 Hz to 10 Hz. Through these operations, the various phases of the evoked intrinsic signal due to the hemodynamic changes of the brain were mapped with a better SNR. Finally, the time series obtained from iterative experiments were averaged to reduce the remaining possible noises and to increase the SNR of the obtained results. The time series related to the hemodynamic intrinsic signals evoked by whisker stimulation before and after applying post-processing analyses in the motor and somatosensory cortices are shown in both healthy and stroke hemispheres (Fig. 1(a-d)). Furthermore, to find the frequency distribution of the OISi data, the PSD for the processed and raw time series were calculated using the Welch function as shown in Fig. 1(e–h).

#### Measuring the characteristics of the brain responses by evaluating the OISi data

In this study, to compare the characteristics of the intrinsic signals such as signal amplitude, signal latency, and the size of the stimulated area, the time series of the 15 s stimulation epochs were used. The characteristics of the intrinsic signals in the motor and somatosensory cortices of the two groups of rats were compared by stimulating contralateral whiskers. Based on the obtained signals which correspond to the literature^2^, the intrinsic signals of the brain are tri-phasic: the initial dip phase, the undershoot phase, and the overshoot phase. In this section, the latency of the brain response to stimulation as well as the amplitude of the signals obtained in the previous section were measured.

The size of the area of the cortex which responded to stimulation can be calculated by employing a ‘pattern detection algorithm’. Considering the results of Chen-Bee *et al*.^2^, in the current study, the time series that correctly represented the behavior of the stimulus-evoked intrinsic signal were considered as the input of the ‘pattern detection algorithm’. This input was called the ‘expected response’. In selecting the ‘expected response’, the numerical temporal specifications of the intrinsic signals measured by Chen-Bee *et al*.^2^ for rats in response to whisker stimulation were used. The convergence value between the characteristics of the experimental time series and those obtained by Chen-Bee *et al*.^2^ was measured. Among the experimental time series, the one with the highest convergence (considering all of the temporal features of the intrinsic signals) was considered as the ‘expected response’. These evaluations were performed separately in both the motor and somatosensory cortices and in both healthy and stroke hemispheres to determine the ‘expected responses’ in these areas. To reduce the calculation volume, the images were segmented into some blocks including 10 × 10 pixels, and the calculations were performed for them rather than the pixels (in the motor and the somatosensory cortices, the size of each block is corresponded to a 100 × 100 μm^2^ area of the cortex). Then, the ‘pattern detection algorithm’ examined the similarity of the response of each block with the input of the algorithm by calculating the Pearson correlation between them. The results are numbers between 0 and 1 and it was assumed that the area with a similarity index ranging from 0.7 to 1 indicates more similarity and represents the area of the cortex activated by stimulation. To obtain the suitable threshold value in the ‘pattern detection’ algorithm, we compared the similarity matrices obtained for different threshold values including 0.5, 0.6, 0.7, 0.8, and 0.9 with the FC matrix of the cortex. It should be noted that to calculate the FC matrix, according to the rat Paxinos atlas^27^, seed location was selected within the area expected to respond to the stimulation of C1-3 and D1-3 whiskers. Afterward, to select the best threshold in this algorithm, the Pearson correlation was measured between the similarity and FC matrices on a component-to-component basis and then the average of the correlation coefficients was calculated across all the components of the matrix. The values of the averaged correlation coefficients for various threshold values are shown in Supplementary Table S9. The results showed that the highest correlation value was obtained for the threshold value of about 0.7 (Supplementary Table S9). In other words, the highest convergence between the similarity matrix and the FC matrix was obtained by choosing 0.7 as the threshold value. The whole size of the area which responded to the stimulus was calculated by summing the areas of the activated blocks.

#### Measuring the correlation-based FC

It should be noted that in measuring the FC of the cortex, Pearson correlation was calculated for both the resting state (300 s control trials) and the stimulus state (315 s stimulus-related trials). At first, as a pre-processing analysis before measuring the FC, a conventional regression method in the fMRI, known as “tCompCorr” global linear model analysis^71^, was used to better interpret the correlations and non-correlations in the FC maps. Then, in order to obtain the correlation-based FC of the cortex, the Pearson correlation between the cortex regions was measured. To reduce the calculation volume, the images were segmented into blocks of 10 × 10 pixels and a time-related signal was assigned to each block by averaging its pixel values. Then, a band pass filter in the frequency range of 0.009 × 0.08 Hz was applied to the signals^3,72–74^. The resulting signal was then used to measure the Pearson correlation between the blocks for evaluating the FC^72,75^. Finally, the Fisher z-transformation was applied to the correlation coefficients^72^ and then these coefficients were normalized between 0 and 1 in accordance with Peri’s study^76^ to obtain the limited and normalized coefficients.

#### Measuring the wavelet-based FC

As in the Pearson correlation-based FC, wavelet-based FC for the resting state and the stimulus state were calculated separately (using 300 s control trials and 315 s stimulus trials, respectively). To measure the wavelet-based FC, each of the acquired images was divided into 12 blocks. Then, the wavelet coherences (WC) between the blocks’ time-series were calculated.

To calculate the WC, the Morlet wavelet function^23^ was used which is defined as:

2 $${{\rm{\psi }}}_{0}(\eta )={\pi }^{-1/4}{e}^{i{\omega }_{0}\eta }{e}^{-{\eta }^{2}/2}$$

where η and ω_0_ indicate the time and frequency of the signal, respectively, and are both dimensionless. In this study, ω_0_ = 6.

To calculate the cross-wavelet and wavelet coherence, the continuous wavelet transforms (CWT) of the time-series must be calculated. In fact, by calculating the CWT, the time-series will be expanded in the time-frequency domain. The CWT of the time-series *M*_n_, with N time points and intervals of δt, is obtained by calculating its convolution with the scaled normalized wavelet functions^23,77,78^. For a faster analysis, however, it is better to perform these calculations in the Fourier space. Hence, the discrete Fourier transforms (DFT) of the time-series and the Fourier transform of the wavelet function are calculated and then the CWT is obtained from the inverse Fourier transform of the product of these two parameters as^23^:

3 $${W}_{n}^{X}(s)=\mathop{\sum }\limits_{k=0}^{N-1}{\hat{M}}_{k}{\hat{\psi }}^{\ast }(s{\omega }_{k}){e}^{i{\omega }_{k}n\delta t}$$

where

4 $${\hat{M}}_{k}=\frac{1}{N}\mathop{\sum }\limits_{n=0}^{N-1}{M}_{n}{e}^{-2\pi ikn/N}$$

and

5 $$\hat{\psi }(s\omega )=\frac{1}{\sqrt{2\pi }}{\int }_{-\infty }^{+\infty }\psi (t/s){e}^{-i\omega t}d(t/s)$$

here k, ω_k_, N, and s are the frequency index, the angular frequency, the number of the time points, and the wavelet scale, respectively. After choosing the wavelet function, it is necessary to calculate CWT in a series of wavelet scales for each time series^23,79^. To compare CWTs at different scales and time-series, it is necessary to normalize the wavelet functions as follows^23,78^:

6 $$\hat{\psi }(s{\omega }_{k})={\left(\frac{2\pi s}{\delta t}\right)}^{1/2}{\hat{\psi }}_{0}(s{\omega }_{k})$$

In order to obtain the phase relationship between the two time-series, the cross wavelet transform (XWT) of the two time-series *M*_n_ and *N*_n_ were calculated by the following equation^79^:

7 $${W}^{MN}={W}^{M}{W}^{N\ast }$$

where power is equal to |*W*_MN_| and phase is equal to arg(*W*_MN_) indicating the relative phase between the two time-series.

Finally, the WC of the two time-series *M*_n_ and *N*_n_ was calculated to obtain the local correlation between the two CWTs by the following equation^23,79^:

8 $${R}_{n}^{2}(s)=\frac{{|S({s}^{-1}{W}_{n}^{MN}(s))|}^{2}}{S({s}^{-1}{|{W}_{n}^{M}(s)|}^{2}).S({s}^{-1}{|{W}_{n}^{N}(s)|}^{2})}$$

here s and S are the wavelet scale and the smoothing operator, respectively. The smoothing operator is defined as^23,79^:

9 $$S(W)={S}_{scale}({S}_{time}({W}_{n}(s)))$$

At first, the wavelet transform is smoothed in time and then the result is smoothed along the axis of the wavelet scale^23,80^.

### Statistical analysis

After analyzing the acquired images, the signal amplitude, the latency of response to stimulation, the size of the activated regions of the cortex, and the FC of the cortex in the healthy and stroke hemispheres were compared using the independent-sample t-test as well as one-way and two-way ANOVA analyses. The data are reported in Supplementary Tables S3–7. P < 0.05 was considered statistically significant.

## Supplementary information

Supplementary information.

Supplementary information 2.

## Data Availability

The data that support the findings of this study are available from the corresponding author on request.
