# Supplementary material for: Measuring the Frequency-Specific Functional Connectivity Using Wavelet Coherence Analysis in Stroke Rats Based on Intrinsic Signals
Source: Sci Rep. 2020 Jun 10;10:9429. doi: 10.1038/s41598-020-66246-9 (PMC7286921; doi:10.1038/s41598-020-66246-9)
Supplement: Supplementary file 1 — Supplementary information. [file 41598_2020_66246_MOESM1_ESM.docx]

**Measuring the Frequency-Specific Functional Connectivity Using Wavelet Coherence Analysis in Stroke Rats Based on Intrinsic Signals**

**Leila Mohammadzadeh1, Hamid Latifi1,2,∗, Sepideh Khaksar3, Mohammad-Sadegh Feiz1, Fereshteh Motamedi4, Amir Asadollahi 1, and Marzieh Ezzatpour2**

*1*Laser and Plasma Research Institute, Shahid Beheshti University, Tehran, 1983969411, Iran

*2*Department of Physics, Shahid Beheshti University, Tehran, 1983963113, Iran

*3*Department of Plant Science, Faculty of Biological Sciences, Alzahra University, Tehran, 1993893973, Iran

*4*Neuroscience Research Center, Shahid Beheshti University of Medical Science, Tehran, 1983963113, Iran

*corresponding author. latifi@sbu.ac.ir

**Supplementary Figures:**


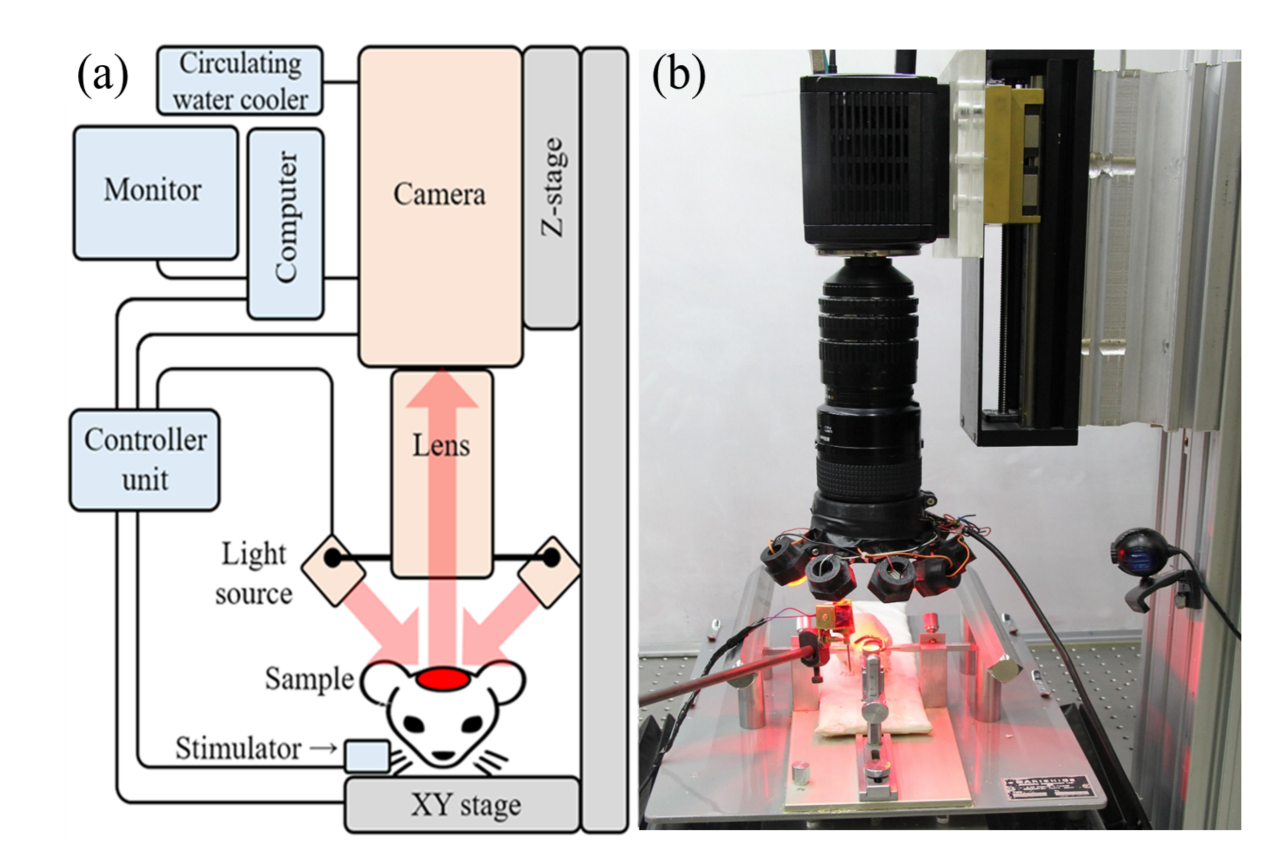


Supplementary Figure S 1: (a). The schematic of the required setup, and (b). the prepared experimental setup for OISi experiments.


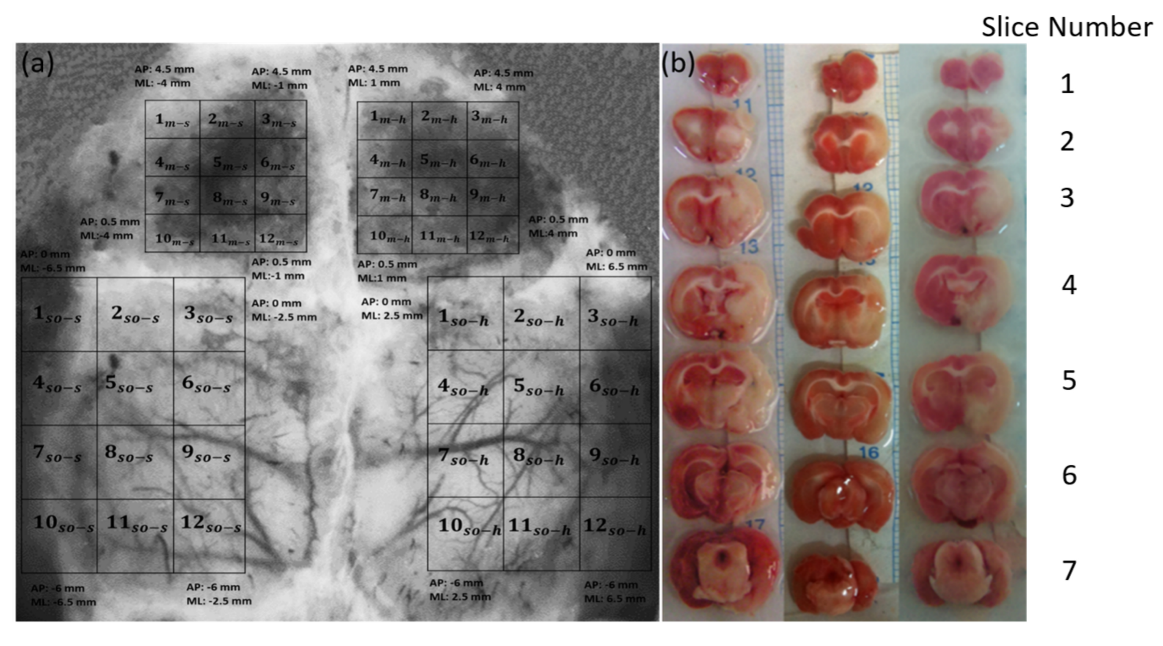


Supplementary Figure S 2: (a). The topographic image of the cortex surface with spatial coordinates and the classification of each area in order to calculate the wavelet coherence. The subscripts m, so, h, and s refer to the motor cortex, the somatosensory cortex, the healthy hemisphere, and the stroke hemisphere, respectively. b. Seven sections of TTC staining of three rat brains in the MCAO group. The white areas are the infarcted areas.


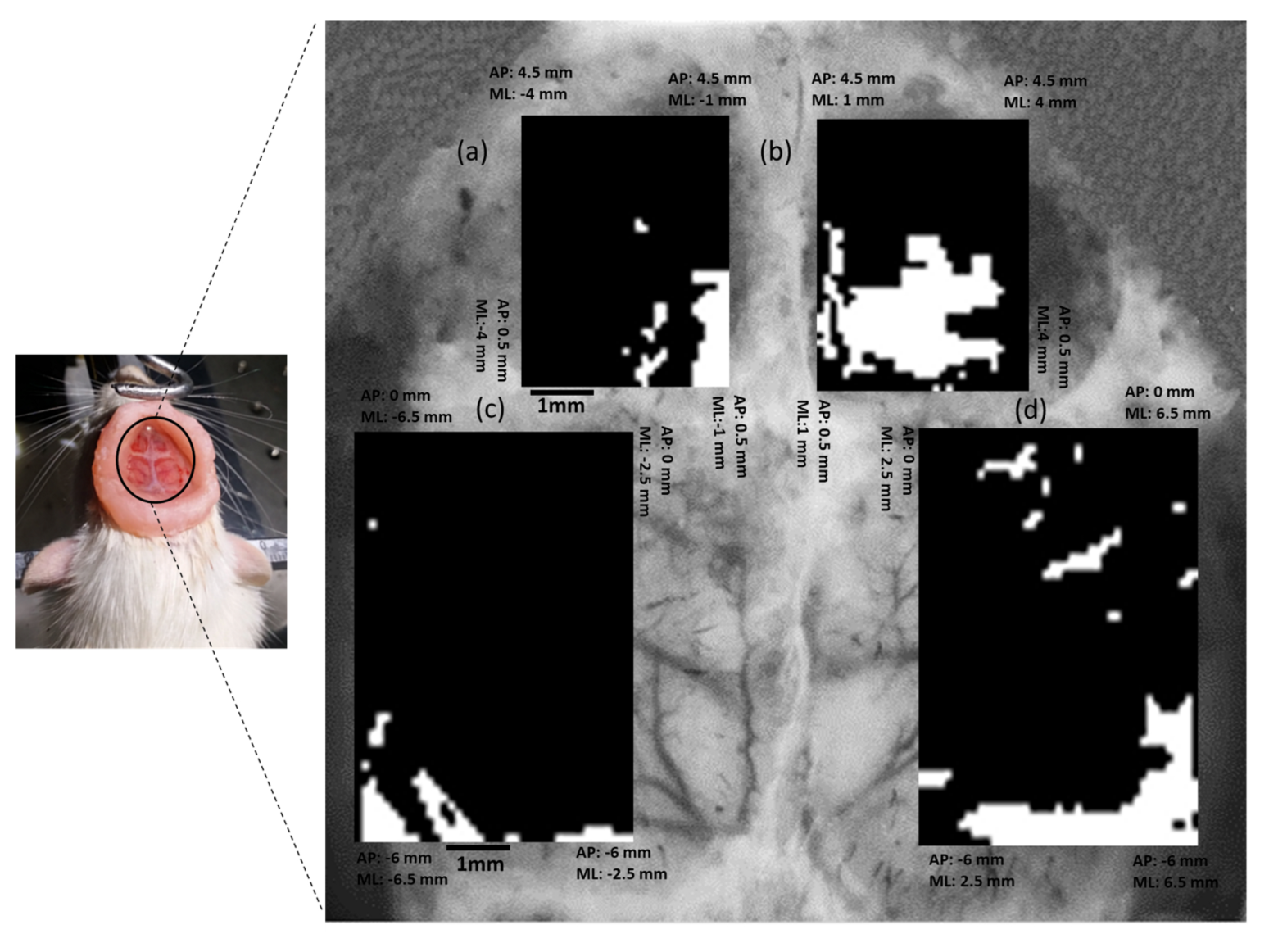


Supplementary Figure S 3: The comparison between the spatial extent of the intrinsic response evoked by stimulating contralateral whiskers is shown in the motor (a and b) and somatosensory (c and d) cortices. a and c are related to the ischemic hemisphere and b and d are related to the healthy hemisphere. The data were thresholded for the similarity indices of 0.7 and higher. The regions in white color illustrate the areas activated in response to stimulation and correspond to the regions with similarity indices of 0.7 and higher. The calculations are done for every 10×10-pixel block and the coordinates of the brain areas under study and the spatial scale are shown in this figure.

**Supplementary Tables:**

Supplementary Table S 1:Neurological Examination after Middle Cerebral Artery Occlusion^1^.

| **Sign** | **Description** | **Score** |
| --- | --- | --- |
| **Postural signs**   1. **Forelimb Flexion** 2. **Thorax Rotation** | **Degree of limb flexion when held by tail**  **Degree of body rotation when held by tail** | **0–2**  **0–2** |
| **Biased movement to pulling the tail three times**  **Biased movement to pushing back of body three times** | **0 or 1 movement toward paretic side**    **Two or three times movement toward paretic side**  **0 or 1 movement toward paretic side**  **Two or three times movement toward paretic side placing of limbs when reaching toward solid surface** | **0**  **1-2**  **0**  **1-2** |
| **Limb placing**   1. **Forelimb** 2. **Hindlimb** | **Normal, weak, or no placing**  **Normal, weak, or no placing** | **0-2**  **0-2** |
| **Symmetry of muscle tone, strength**   1. **Lateral resistance** 2. **Grasping strength** | **Degree of resistance against lateral push**  **Symmetry of grasping strength onto wire cage** | **0-2**  **0-1** |
| **Sensory function**   1. **Grasping reflex of forepaw** 2. **Touching reflex** | **Grasping onto tube when gently touched 0–1**  **Withdrawal of forelimb when touched by needle** | **0-1**  **0-1** |
| **Gait Disturbance** | **Straight walking**  **Walking towards contralateral side**  **Alternate circling and walking straight**  **Alternate circling and walking towards paretic side**  **Circling and/or other gait disturbance (backing, crawling, walking on digits)**  **Constant circling toward paretic side** | **0**  **1**  **2**  **3**  **4**  **5** |
| **Climbing** | **Climbing up an inclined board (45◦)** | **0-1** |
| **Motility, spontaneous activity** | **Animal observed for 1 min**  **Normal or slightly reduced exploratory behavior Moving limbs without proceeding**  **Moving only to stimuli**  **Unresponsive to stimuli, with normal muscle tone**  **Severely decreased tone, premortal signs** | **0-1**  **2**  **3**  **4**  **5** |

Supplementary Table S 2: The neurologic deficits scores in control and middle cerebral artery occlusion (MCAO) groups. This table indicates partial scores of rats’ sensory and motor capabilities (n=6 in each group). Detail information about this tests explained in Supplementary Table S1.

| Groups | | MCAO | | | | | | Control | | | | | |
| --- | --- | --- | --- | --- | --- | --- | --- | --- | --- | --- | --- | --- | --- |
| Rat # | | 1 | 2 | 3 | 4 | 5 | 6 | 1 | 2 | 3 | 4 | 5 | 6 |
| Postural Signs | **Thorax Twisting** | 2 | 2 | 2 | 2 | 2 | 2 | 0 | 0 | 0 | 0 | 0 | 0 |
|  | **Forelimb Flexion** | 2 | 2 | 2 | 2 | 2 | 2 | 0 | 0 | 0 | 0 | 0 | 0 |
| Biased Movement | **Pulling** | 1 | 1 | 2 | 2 | 2 | 2 | 0 | 0 | 0 | 0 | 0 | 0 |
|  | **Pushing Back** | 2 | 1 | 2 | 2 | 1 | 2 | 1 | 0 | 1 | 1 | 1 | 0 |
| Limb Placing | **Forelimb** | 2 | 1 | 2 | 2 | 2 | 1 | 0 | 0 | 0 | 0 | 0 | 0 |
|  | **Hind limb** | 2 | 1 | 2 | 2 | 2 | 2 | 0 | 0 | 0 | 0 | 1 | 1 |
| Symmetry of Muscle Tone | **Lateral Resistance** | 1 | 1 | 1 | 1 | 1 | 1 | 0 | 1 | 0 | 0 | 0 | 1 |
|  | **Grasping Strength** | 1 | 1 | 2 | 1 | 2 | 2 | 1 | 0 | 1 | 0 | 0 | 0 |
| Sensory Function | **Grasping Reflex** | 1 | 0 | 1 | 1 | 1 | 1 | 0 | 0 | 0 | 0 | 0 | 0 |
|  | **Touching Reflex** | 1 | 1 | 1 | 1 | 1 | 1 | 0 | 0 | 0 | 0 | 0 | 0 |
| Gait Disturbances | | 5 | 5 | 4 | 4 | 3 | 5 | 1 | 0 | 1 | 0 | 0 | 1 |
| Climbing | | 1 | 1 | 1 | 1 | 0 | 1 | 0 | 0 | 0 | 0 | 1 | 0 |
| Motility | | 4 | 4 | 3 | 4 | 4 | 4 | 1 | 0 | 0 | 0 | 1 | 1 |
| Sum | | 25 | 21 | 25 | 25 | 23 | 26 | 4 | 1 | 3 | 1 | 4 | 4 |
| Average | | **24.17** | | | | | | **2.83** | | | | | |

Supplementary Table S 3: The results of the independent sample t-test analysis for comparing the cortical response, FC, and WC between the healthy and stroke motor and somatosensory cortices in the MCAO group.

| Parameter | Cortex | Hemisphere | Mean value | SEM | *P*- value |
| --- | --- | --- | --- | --- | --- |
| Signal amplitude | Motor | Healthy | 0.008 | ± 0.0002 | 2.7E-11 |
|  |  | Stroke | 0.003 | ± 0.00005 |  |
|  | Somatosensory | Healthy | 0.005 | ± 0.0002 | 1.7E-18 |
|  |  | Stroke | 0.002 | ± 0.00007 |  |
| Latency of the signal | Motor | Healthy | 0.39 s | ± 0.02 s | 2.7E-15 |
|  |  | Stroke | 1.30 s | ± 0.06 s |  |
|  | Somatosensory | Healthy | 0.35 s | ± 0.01 s | 9.2E-7 |
|  |  | Stroke | 1.34 s | ± 0.02 s |  |
| Size of the responded area | Motor | Healthy | 1.85 mm2 | ± 0.05 mm2 | 2.4E-10 |
|  |  | Stroke | 0.51 mm2 | ± 0.05 mm2 |  |
|  | Somatosensory | Healthy | 2.91 mm2 | ± 0.11mm2 | 2.7E-9 |
|  |  | Stroke | 0.58 mm2 | ± 0.10 mm2 |  |
| Resting-state correlation strength | Motor | Healthy | 0.54 | ± 0.01 | 0.0009 |
|  |  | Stroke | 0.37 | ± 0.02 |  |
|  | Somatosensory | Healthy | 0.55 | ± 0.003 | 7.5E-8 |
|  |  | Stroke | 0.43 | ± 0.008 |  |
| Stimulus-related correlation strength | Motor | Healthy | 0.51 | ± 0.02 | 0.006 |
|  |  | Stroke | 0.36 | ± 0.03 |  |
|  | Somatosensory | Healthy | 0.52 | ± 0.01 | 8.3E-7 |
|  |  | Stroke | 0.42 | ± 0.01 |  |
| resting-state wavelet coherence | Motor | Healthy | 0.47 | ± 0.002 | 2.8E-25 |
|  |  | Stroke | 0.45 | ± 0.002 |  |
|  | Somatosensory | Healthy | 0.54 | ± 0.003 | 0.002 |
|  |  | Stroke | 0.52 | ± 0.002 |  |
| stimulus-related wavelet coherence | Motor | Healthy | 0.54 | ± 0.003 | 3.0E-109 |
|  |  | Stroke | 0.45 | ± 0.001 |  |
|  | Somatosensory | Healthy | 0.49 | ± 0.002 | 1.0E-25 |
|  |  | Stroke | 0.45 | ± 0.0014 |  |

Supplementary Table S 4: The statistical comparison of the WC values in the 4 frequency ranges between the healthy and stroke motor cortices in the MCAO group. The negative sign in the percentage of the difference rate indicates a decrease the WC value in the stroke hemisphere.

| frequency range | Surface area percentage with significant difference | Percentage of the difference rate | *P*-value |
| --- | --- | --- | --- |
| $\mathbf{I}$ | 47% | -18% | 8.76E-71 |
| $\mathbf{II}$ | 58% | -28% | 5.4E-204 |
| $\mathbf{III}$ | 37% | -11% | 6.84E-32 |
| $\mathbf{IV}$ | 34% | -12% | 1.24E-66 |

Supplementary Table S 5: The statistical comparison of the WC values in the 4 frequency ranges between the healthy and stroke somatosensory cortices in the MCAO group. The negative sign in the percentage of the difference rate indicates a decrease of the WC value in the stroke hemisphere.

| frequency range | Surface area percentage with significant difference | Percentage of the difference rate | *P*-value |
| --- | --- | --- | --- |
| $\mathbf{I}$ | 49% | -6% | 9.75E-10 |
| $\mathbf{II}$ | 44% | -5% | 4.33E-7 |
| $\mathbf{III}$ | 34% | -12% | 1.12E-51 |
| $\mathbf{IV}$ | 26% | -11% | 1.06E-50 |

Supplementary Table S 6: The statistical comparison of the WC values in the 16 time-frequency domains between the healthy and stroke motor cortices in the MCAO group. The negative sign in the percentage of the difference rate indicates a decrease of the WC value in the stroke hemisphere.

| Time–frequency domain | Surface area percentage with significant difference | Percentage of the difference rate | *P*-value |
| --- | --- | --- | --- |
| $\mathbf{I}\boldsymbol{-\Delta}\boldsymbol{t}_{\boldsymbol{1}}$ | 24% | -16% | 5.52E-4 |
| $\mathbf{I}\boldsymbol{-\Delta}\boldsymbol{t}_{\boldsymbol{2}}$ | 52% | -21% | 1.29E-11 |
| $\mathbf{I}\boldsymbol{-\Delta}\boldsymbol{t}_{\boldsymbol{3}}$ | 56% | -17% | 6.30E-5 |
| $\mathbf{I}\boldsymbol{-\Delta}\boldsymbol{t}_{\boldsymbol{4}}$ | 58% | -18% | 5.00E-6 |
| $\mathbf{II}\boldsymbol{-\Delta}\boldsymbol{t}_{\boldsymbol{1}}$ | 62% | -30% | 1.38E-49 |
| $\mathbf{II}\boldsymbol{-\Delta}\boldsymbol{t}_{\boldsymbol{2}}$ | 48% | -28% | 6.97E-36 |
| $\mathbf{II}\boldsymbol{-\Delta}\boldsymbol{t}_{\boldsymbol{3}}$ | 60% | -25% | 1.73E-21 |
| $\mathbf{II}\boldsymbol{-\Delta}\boldsymbol{t}_{\boldsymbol{4}}$ | 62% | -27% | 1.10E-32 |
| $\mathbf{III}\boldsymbol{-\Delta}\boldsymbol{t}_{\boldsymbol{1}}$ | 38% | -17% | 9.50E-08 |
| $\mathbf{III}\boldsymbol{-\Delta}\boldsymbol{t}_{\boldsymbol{2}}$ | 32% | -7% | 0.96 |
| $\mathbf{III}\boldsymbol{-\Delta}\boldsymbol{t}_{\boldsymbol{3}}$ | 42% | -3% | 0.69 |
| $\mathbf{III}\boldsymbol{-\Delta}\boldsymbol{t}_{\boldsymbol{4}}$ | 36% | -9% | 0.86 |
| $\mathbf{IV}\boldsymbol{-\Delta}\boldsymbol{t}_{\boldsymbol{1}}$ | 56% | -22% | 4.79E-53 |
| $\mathbf{IV}\boldsymbol{-\Delta}\boldsymbol{t}_{\boldsymbol{2}}$ | 26% | -13% | 1.24E-08 |
| $\mathbf{IV}\boldsymbol{-\Delta}\boldsymbol{t}_{\boldsymbol{3}}$ | 21% | -4% | 0.99 |
| $\mathbf{IV}\boldsymbol{-\Delta}\boldsymbol{t}_{\boldsymbol{4}}$ | 32% | -6% | 0.99 |

Supplementary Table S 7: The statistical comparison of the WC values in the 16 time-frequency domains between the healthy and stroke somatosensory cortices in the MCAO group. The negative sign in the percentage of the difference rate indicates a decrease of the WC value in the stroke hemisphere.

| Time-frequency domain | Surface area percentage with significant difference | Percentage of the difference rate | *P*-value |
| --- | --- | --- | --- |
| $\mathbf{I}\boldsymbol{-\Delta}\boldsymbol{t}_{\boldsymbol{1}}$ | 30% | -8% | 4.80E-5 |
| $\mathbf{I}\boldsymbol{-\Delta}\boldsymbol{t}_{\boldsymbol{2}}$ | 47% | -4% | 0.04 |
| $\mathbf{I}\boldsymbol{-\Delta}\boldsymbol{t}_{\boldsymbol{3}}$ | 59% | -5% | 4.58E-3 |
| $\mathbf{I}\boldsymbol{-\Delta}\boldsymbol{t}_{\boldsymbol{4}}$ | 60% | -6% | 8.45E-4 |
| $\mathbf{II}\boldsymbol{-\Delta}\boldsymbol{t}_{\boldsymbol{1}}$ | 32% | -10% | 6.61E-09 |
| $\mathbf{II}\boldsymbol{-\Delta}\boldsymbol{t}_{\boldsymbol{2}}$ | 35% | -4% | 0.02 |
| $\mathbf{II}\boldsymbol{-\Delta}\boldsymbol{t}_{\boldsymbol{3}}$ | 65% | -2% | 0.32 |
| $\mathbf{II}\boldsymbol{-\Delta}\boldsymbol{t}_{\boldsymbol{4}}$ | 45% | -2% | 0.28 |
| $\mathbf{III}\boldsymbol{-\Delta}\boldsymbol{t}_{\boldsymbol{1}}$ | 32% | -11% | 1.11E-12 |
| $\mathbf{III}\boldsymbol{-\Delta}\boldsymbol{t}_{\boldsymbol{2}}$ | 17% | -2% | 1.68E-01 |
| $\mathbf{III}\boldsymbol{-\Delta}\boldsymbol{t}_{\boldsymbol{3}}$ | 41% | -16% | 3.19E-26 |
| $\mathbf{III}\boldsymbol{-\Delta}\boldsymbol{t}_{\boldsymbol{4}}$ | 48% | -17% | 3.38E-30 |
| $\mathbf{IV}\boldsymbol{-\Delta}\boldsymbol{t}_{\boldsymbol{1}}$ | 18% | -11% | 4.28E-19 |
| $\mathbf{IV}\boldsymbol{-\Delta}\boldsymbol{t}_{\boldsymbol{2}}$ | 21% | -11% | 8.33E-18 |
| $\mathbf{IV}\boldsymbol{-\Delta}\boldsymbol{t}_{\boldsymbol{3}}$ | 24% | -7% | 9.40E-07 |
| $\mathbf{IV}\boldsymbol{-\Delta}\boldsymbol{t}_{\boldsymbol{4}}$ | 39% | -13% | 1.51E-24 |

Supplementary Table S 8: Infarction volumes in the MCAO group of rats using TTC staining in mm^3^.

| Rat# | 1 | 2 | 3 | 4 | 5 | 6 |
| --- | --- | --- | --- | --- | --- | --- |
| Infarction Volume (mm^3^) | 211.1232 mm^3^ | 214.1332 mm^3^ | 184.4824 mm^3^ | 179.3105 mm^3^ | 195.346 mm^3^ | 233.1045 mm^3^ |

Supplementary Table S 9: Convergence values between similarity and FC matrices for different threshold values. The mean and the standard error of the mean (SEM) of the average convergence were calculated between several trials.

| Threshold | 0.5 | 0.6 | 0.7 | 0.8 | 0.9 |
| --- | --- | --- | --- | --- | --- |
| Mean of convergence | 0.46 | 0.63 | 0.86 | 0.71 | 0.58 |
| SEM of convergence | 0.043 | 0.011 | 0.017 | 0.032 | 0.007 |

**References**

1 Reglődi, D., Tamas, A. & Lengvari, I. Examination of sensorimotor performance following middle cerebral artery occlusion in rats. *Brain research bulletin* **59**, 459-466 (2003).
