# Supplementary material for: Measuring the Frequency-Specific Functional Connectivity Using Wavelet Coherence Analysis in Stroke Rats Based on Intrinsic Signals
Source: Sci Rep. 2020 Jun 10;10:9429. doi: 10.1038/s41598-020-66246-9 (PMC7286921; doi:10.1038/s41598-020-66246-9)
Supplement: Supplementary file 2 — Supplementary information 2. [file 41598_2020_66246_MOESM2_ESM.docx]

**Figure 1:**a-d. Hemodynamic intrinsic signal evoked by whisker stimulation for (a) healthy motor , (b) stroke motor, (c) healthy somatosensory, and(d) stroke somatosensory cortices. The signals for raw data and signals after pre-processing analysis are shown in red and blue colors, respectively. e-h. PSD for (e) healthy motor, (f) stroke motor, (g) healthy somatosensory, and (h) stroke somatosensory cortices, related to (a-d) panels, respectively. PSD for raw data and data after pre-processing analysis are shown in red and blue colors, respectively.

**Figure 2:** a-b. The comparison of the signal amplitude and the latency of the intrinsic signals evoked by stimulating contralateral whiskers in the ischemic and healthy hemispheres. The yellow rectangle and colored bars show the stimulation period and the latency of the responses, respectively. The latency of signal in healthy and stroke hemisphere are shown in blue and red bars, respectively. The comparison between the spatial extent of the intrinsic response evoked by stimulating contralateral whiskers is shown in the motor (c and d) and somatosensory (e and f) cortices. c and e are related to the stroke hemisphere and d and f are related to the healthy hemisphere. The regions in red color illustrate the areas activated in response to stimulation. The calculations are done for every 10×10-pixel block and the coordinates of the brain areas under study and the spatial scale are shown in this figure. The waveforms of the expected responses used in the ‘pattern detection algorithm’ in the panels (c-f) are shown in the upper left corners of these panels.

**Figure 3:** The comparison of the XWT and WC maps of the healthy and ischemic motor cortices. (a) Healthy and (f) ischemic motor cortices. Two randomly selected blocks in both hemispheres are displayed by white arrows. b and g are the time series of each selected block and are related to a and f, respectively. c and h are PSD of each selected block and are related to a and f, respectively. d and i are the XWT maps of the selected blocks in healthy and ischemic motor cortices, respectively. The relative phase between two time-series is displayed by arrows in these plots. e and j are the WC maps of the selected blocks in healthy and ischemic motor cortices, respectively. The color bars in WC maps show the WC power values that indicate the areas in the time-frequency domain where two time-series co-vary.

**Figure 4:** The averaged WC values in the 16 time-frequency domains in the motor (a and b) and somatosensory (c and d) cortices. Figures a and c illustrate relatively similar maps whereas Figures b and d illustrate different maps. The blocks are selected symmetrically in the healthy and stroke hemispheres.

**Figure 5:** The statistical matrixes for the 16 time-frequency domains. The elements of the matrixes indicate the P-values of the two-way ANOVA analyses between stimulus-related WCs in healthy and stroke hemispheres. The red, orange, yellow, blue, and white colors show the P-values of < 0.001, < 0.01, < 0.05, ≥0.05, and 0, respectively.

**Figure 6:** The mean WC value for the 16 time-frequency domains in the healthy and ischemic hemispheres averaged across all blocks within the motor cortex. The mean and the standard error of the mean of the average WC values at all time-frequency domains are illustrated in this figure. The P value of the ANOVA analysis is written on each related graph. ** P<0.01; # P<0.00001.

**Figure 7:** The comparison of the average WC values in the specified frequencies between the healthy and stroke hemispheres in (a) the motor cortex and in (b) the somatosensory cortex. The P value of the ANOVA analysis is written on each related graph. * P<0.05; ** P<0.01; *** P<0.001; **** P<0.0001; # P<0.00001; n.s. P≥ 0.05.

**Supplementary Figure S 1:** (a). The schematic of the required setup, and (b). the prepared experimental setup for OISi experiments.

**Supplementary Figure S 2:** (a). The topographic image of the cortex surface with spatial coordinates and the classification of each area in order to calculate the wavelet coherence. The subscripts m, so, h, and s refer to the motor cortex, the somatosensory cortex, the healthy hemisphere, and the stroke hemisphere, respectively. b. Seven sections of TTC staining of three rat brains in the MCAO group. The white areas are the infarcted areas.

**Supplementary Figure S 3:** The comparison between the spatial extent of the intrinsic response evoked by stimulating contralateral whiskers is shown in the motor (a and b) and somatosensory (c and d) cortices. a and c are related to the ischemic hemisphere and b and d are related to the healthy hemisphere. The data were thresholded for the similarity indices of 0.7 and higher. The regions in white color illustrate the areas activated in response to stimulation and correspond to the regions with similarity indices of 0.7 and higher. The calculations are done for every 10×10-pixel block and the coordinates of the brain areas under study and the spatial scale are shown in this figure.

**Supplementary Table S 1:**Neurological Examination after Middle Cerebral Artery Occlusion^1^

**Supplementary Table S 2:** The neurologic deficits scores in control and middle cerebral artery occlusion (MCAO) groups. This table indicates partial scores of rats’ sensory and motor capabilities (n=6 in each group). Detail information about this tests explained in Supplementary Table S1.

**Supplementary Table S 3:** The results of the independent sample t-test analysis for comparing the cortical response, FC, and WC between the healthy and stroke motor and somatosensory cortices in the MCAO group.

**Supplementary Table S 4:** The statistical comparison of the WC values in the 4 frequency ranges between the healthy and stroke motor cortices in the MCAO group. The negative sign in the percentage of the difference rate indicates a decrease the WC value in the stroke hemisphere.

**Supplementary Table S 5:** The statistical comparison of the WC values in the 4 frequency ranges between the healthy and stroke somatosensory cortices in the MCAO group. The negative sign in the percentage of the difference rate indicates a decrease of the WC value in the stroke hemisphere.

**Supplementary Table S 6:** The statistical comparison of the WC values in the 16 time-frequency domains between the healthy and stroke motor cortices in the MCAO group. The negative sign in the percentage of the difference rate indicates a decrease of the WC value in the stroke hemisphere.

**Supplementary Table S 7:** The statistical comparison of the WC values in the 16 time-frequency domains between the healthy and stroke somatosensory cortices in the MCAO group. The negative sign in the percentage of the difference rate indicates a decrease of the WC value in the stroke hemisphere.

**Supplementary Table S 8:** Infarction volumes in the MCAO group of rats using TTC staining in mm^3^.

**Supplementary Table S 9:** Convergence values between similarity and FC matrices for different threshold values. The mean and the standard error of the mean (SEM) of the average convergence were calculated between several trials.

**References**

1 Reglődi, D., Tamas, A. & Lengvari, I. Examination of sensorimotor performance following middle cerebral artery occlusion in rats. Brain research bulletin **59**, 459-466 (2003).
